# YTHDF1-enhanced iron metabolism depends on TFRC m^6^A methylation

**DOI:** 10.7150/thno.51231

**Published:** 2020-10-26

**Authors:** Jing Ye, Zhanggui Wang, Xiaozhen Chen, Xiaohua Jiang, Zhihuai Dong, Sunhong Hu, Wenya Li, Yuehui Liu, Bing Liao, Weidong Han, Jiaying Shen, Mang Xiao

**Affiliations:** 1 Department of Otolaryngology Head and Neck Surgery, Sir Run Run Shaw Hospital, College of Medicine, Zhejiang University, Hangzhou, Zhejiang, China.; 2 Department of Radiotherapy, The Second People's Hospital of Anhui Province, Hefei, Anhui, China.; 3 Laboratory of Cancer Biology, Institute of Clinical Science, Sir Run Run Shaw Hospital, College of Medicine, Zhejiang University, Hangzhou, Zhejiang, China.; 4 Department of Otorhinolaryngology Head and Neck Surgery, The Second Affiliated Hospital Of Nanchang University, Nanchang, Jiangxi, China.; 5 Department of Medical Oncology, Sir Run Run Shaw Hospital, College of Medicine, Zhejiang University, Hangzhou, Zhejiang, China.

**Keywords:** Hypopharyngeal squamous cell carcinoma, N6-methyladenosine (m^6^A) modification, YTHDF1, Iron metabolism, TFRC

## Abstract

**Background:** Among head and neck squamous cell carcinomas (HNSCCs), hypopharyngeal squamous cell carcinoma (HPSCC) has the worst prognosis. Iron metabolism, which plays a crucial role in tumor progression, is mainly regulated by alterations to genes and post-transcriptional processes. The recent discovery of the N6-methyladenosine (m^6^A) modification has expanded the realm of previously undiscovered post-transcriptional gene regulation mechanisms in eukaryotes. Many studies have demonstrated that m^6^A methylation represents a distinct layer of epigenetic deregulation in carcinogenesis and tumor proliferation. However, the status of m^6^A modification and iron metabolism in HPSCC remains unknown.

**Methods:** Bioinformatics analysis, sample analysis, and transcriptome sequencing were performed to evaluate the correlation between m^6^A modification and iron metabolism. Iron metabolic and cell biological analyses were conducted to evaluate the effect of the m^6^A reader YTHDF1 on HPSCC proliferation and iron metabolism. Transcriptome-wide m^6^A-seq and RIP-seq data were mapped to explore the molecular mechanism of YTHDF1 function in HPSCC.

**Results:** YTHDF1 was found to be closely associated with ferritin levels and intratumoral iron concentrations in HPSCC patients at Sir Run Run Shaw Hospital. YTHDF1 induced-HPSCC tumorigenesis depends on iron metabolism *in vivo in vitro*. Mechanistically, YTHDF1 methyltransferase domain interacts with the 3'UTR and 5'UTR of TRFC mRNA, then further positively regulates translation of m^6^A-modified TFRC mRNA. Gain-of-function and loss-of-function analyses validated the finding showing that TFRC is a crucial target gene for YTHDF1-mediated increases in iron metabolism.

**Conclusion:** YTHDF1 enhanced TFRC expression in HPSCC through an m^6^A-dependent mechanism. From a therapeutic perspective, targeting YTHDF1 and TFRC-mediated iron metabolism may be a promising strategy for HPSCC.

## Introduction

Head and neck squamous cell carcinoma (HNSCC) is the sixth most common cancer worldwide, with approximately 700,000 cases diagnosed per year 1. Of all the areas affected (oral, oropharyngeal, nasal/paranasal, laryngeal, hypopharyngeal, cervical esophageal locations, etc.), hypopharyngeal squamous cell carcinoma (HPSCC) has the worst prognosis. HPSCC is generally detected at an advanced stage because of a lack of biomarkers for early diagnosis 2,3.

To date, except for EGFR inhibitors, no FDA-approved targeted therapies are available. With current standard multimodality therapies, the 5-year overall survival (OS) rate remains low, varying between 28% and 41% 4,5. Increasing evidence indicates that the unique clinical and biological characteristics of HPSCC are due to anatomic location of the tumor and genetic and transcriptome alterations 6,7. For example, co-occurring CCND1 and CDKN2A mutations, and chromosomal instability markers are associated with chemoradiotherapy outcomes in advanced stage HPSCC patients 8. We found that multiple genomic alterations have been analyzed in relevance of sensitivity to chemotherapy, targeted therapy, and ionizing radiation 9. However, few specific biomarkers and therapeutic targets for HPSCC management have identified and validated. Therefore, a better understanding of the molecular mechanism of HPSCC progression is critical.

Iron plays a crucial role in tumor progression due to both its major contributions to tumor survival and reprogramming of the tumor microenvironment 10. Large-scale epidemiological studies have shown a direct correlation between systemic iron levels and increased cancer risk 11,12. Dysregulation of iron homeostasis typically manifests as upregulation of iron uptake and downregulation of iron efflux 13. First, tumor cells exhibit uncontrolled growth and proliferation and require more iron than do normal cells 14-16. In other words, excessive iron intake by cells is one of the hallmarks of carcinogenesis. Second, cytoplasmic iron is stored mainly as ferritin, which is critical for the maintenance of iron metabolism and protection of cells from oxidative damage 17; however, the levels of iron and ferritin in malignant tumors can be 5~6-fold higher than those in benign tumors 18. Excessive iron may cause redox imbalance and generate reactive oxygen species (ROS) in tumor cells, which increase genomic instability and proliferation 19. Moreover, serum ferritin is also increased in cancer patients compared to healthy individuals 20. Several studies have suggested that the reprogramming of iron metabolism is a major aspect of HNSCC. Multiple iron-related genes are significantly elevated in HNSCC relative to those in normal squamous epithelium, resulting in increased intratumoral iron accumulation and cell proliferation 21,22. To date, there is limited knowledge regarding the molecular regulation of intracellular iron metabolism in HNSCC 22.

The identification and functional characterization of proteins that specifically recognize the m^6^A modification in RNA (RNA m^6^A) revealed that this modification induces cells to accelerate mRNA metabolism and translation. These proteins include methyltransferase complexes, demethylases, and a group of specific RNA-binding proteins, also known as “writers”, “erasers”, and “readers” 23. Writers (such as METTL3/METTL14 complexes) and erasers (such as FTO and ALKBH5) determine the prevalence and distribution of m^6^A. Previous studies demonstrated that the m^6^A modification mediated by writers METTL3 and METTL14 enhanced the stability of LncAROD, thus exacerbating the malignant behavior of HNSCC cells 24. METTL14 also drives Epstein-Barr virus (EBV)-mediated tumorigenesis 25. The m^6^A reader IGF2BP2 was reported to be upregulated in HNSCC tumor tissues, and high IGF2BP2 expression is associated with poor prognosis for patients with HNSCC 26. Readers (such as YTHDFs) primarily recruit proteins that mediate the effects of m^6^A modification on mRNAs 27-29. For YTHDFs, including YTHDF1, YTHDF2 and YTHDF3; YTHDF1, are enriched in the cytoplasm and have been recognized to enhance the translational efficiency of m^6^A-modified mRNAs 30. YTHDF1 expression is amplified in various types of cancers, including HNSCC; furthermore, it has a critical oncogenic role 31-38. Recent findings also showed that YTHDF1-deficient mice show an elevated antigen-specific CD8(+) T cell anti-tumor response 32. YTHDF2 mediates mRNA instability 30,39, while YTHDF3 facilitates translation in conjunction with YTHDF1 and affects the decay of m^6^A-modified mRNAs 40.

Previous studies demonstrated that the m^6^A modification was correlated with carcinogenesis, tumor proliferation and chemoresistance of cancer cells 31,34,41-43. However, the effect of m^6^A modifications on cancer progression depends on whether the m^6^A target gene is an oncogene or a tumor suppressor, the extent of the change in m^6^A level in cancer cells, and, most importantly, the post-modification regulation of target mRNA. Despite the numerous discoveries highlighting the association between m^6^A modification and cancer development, the underlying regulatory mechanism of m^6^A modification in HPSCC, especially with regard to iron metabolism of HPSCC, remains poorly understood. Herein, we investigated the role of the m^6^A modification in HPSCC and identified the oncogenic role of YTHDF1. By thoroughly investigating the role of YTHDF1 deregulation in HPSCC, we found that YTHDF1 is closely associated with tumor proliferation and iron metabolism. Our study illustrates the critical role of YTHDF1 in human HPSCC carcinogenesis and iron transport pathways. These results might shed light on possible clinical therapies for treating HPSCC patients with high iron overload.

## Materials and Methods

### Patient specimens

Two cohorts of HPSCC patients who underwent surgery at individual medical centers between 2012 and 2020 were included in this study.

Tissue specimens from patients in cohort 1 included whole blood, freshly resected cancerous and normal tissues, and paraffin-embedded tissues, all of which obtained from Sir Run Run Shaw Hospital, College of Medicine, Zhejiang University. The blood and excised cancerous and normal tissues were stored and numbered in the Sir Run Run Shaw Hospital biological specimen bank, whereas the paraffin-embedded surgical specimens were permanently stored in the Department of Pathology at Sir Run Run Shaw Hospital.

The tissue specimens from patients in cohort 2 comprised paraffin-embedded surgical specimens, which were obtained from The Second Affiliated Hospital of Nanchang University Medical College.

None of these patients had received radiotherapy or chemotherapy prior to surgery and had no history of hepatic disease.

### Iron assay and intracellular chelatable iron (Fe^2+^) and ROS measurements

Intracellular iron (ferrous iron and ferric iron) levels were determined using an iron assay kit from Sigma-Aldrich (MAK025). Intracellular chelatable iron (Fe^2+^) was measured with the fluorescent indicator Phen Green SK fluorescent probe (#P- 14313, Life Technologies, Grand Island, NY, USA). Levels of ROS were determined using DCFH-DA (Solarbio, D6470) according to the manufacturer's instructions. The detailed protocols are described in the *Supplementary Materials and Methods.*

### m^6^A quantification

The global m^6^A levels in mRNA were measured with an EpiQuik m^6^A RNA methylation quantification kit (Colorimetric) (Epigenetic, Farmingdale, NY) following the manufacturer's protocol.

### RNA-Seq, MeRIP and anti-m^6^A immunoprecipitation (MeRIP-qPCR)

Total RNA was extracted using Trizol reagent (Invitrogen, CA, USA) following the manufacturer's protocol. Eluted m^6^A-containing fragments (IP) and untreated input control fragments were converted into the final cDNA library by the dUTP method in accordance with strand-specific library preparation. The average insert size for the paired-end libraries was ~ 100 ± 50 bp. Then, we performed paired-end 2 × 150 bp sequencing on an Illumina NovaSeq 6000 platform at LC-BIO Bio-tech Ltd. (Hangzhou, China) following the vendor's recommended protocol. Real-time PCR was carried out following m^6^A-IP to quantify the changes in the m^6^A methylation levels of a specific target gene. The detailed MeRIP and MeRIP-qPCR protocols are described in the *Supplementary Materials and Methods.*

### RNA immunoprecipitation (RIP) and high-throughput sequencing

The enrichment of RNA was normalized to the IgG level. cDNA libraries were produced by employing an NEBNext UltraRNA Library Prep Kit for Illumina (New England Biolabs) and sequenced on an Illumina NovaSeq 6000 platform at LC-BIO Bio-tech Ltd. (Hangzhou, China) following the vendor's recommended protocol. RIP was performed using an EZ-Magna RIP RNA-binding protein immunoprecipitation kit (Millipore) following the manufacturer's protocol with some modifications. The detailed protocol is described in the *Supplementary Materials and Methods*.

### Statistical analyses

Bioinformatic analyses, including Gene Ontology (GO), Kyoto Encyclopedia of Genes and Genomes (KEGG), and gene set enrichment analysis (GSEA), were performed using the OmicStudio tools at https://www.omicstudio.cn/tool. All statistical analyses were carried out using GraphPad Prism version 7 (GraphPad Software, CA) for Windows or R software (www.r-project.org). Statistical significance was assessed by unpaired two tailed Student t-tests, analysis of variance (ANOVA) or Spearman rank correlation. Recurrence-free survival was evaluated by the Kaplan-Meier method and log-rank test. The data are expressed as the means ± SD. Statistical significance was indicated as follows: n.s. no significance, *p < 0.05; **p < 0.01; ***p < 0.001, ****p < 0.0001. Each experiment was repeated independently at least three times.

More detailed materials and methods are in the *Supplementary Materials and Methods*.

## Results

### YTHDF1 is closely correlated with iron metabolism in HPSCC

**Figure 1:**
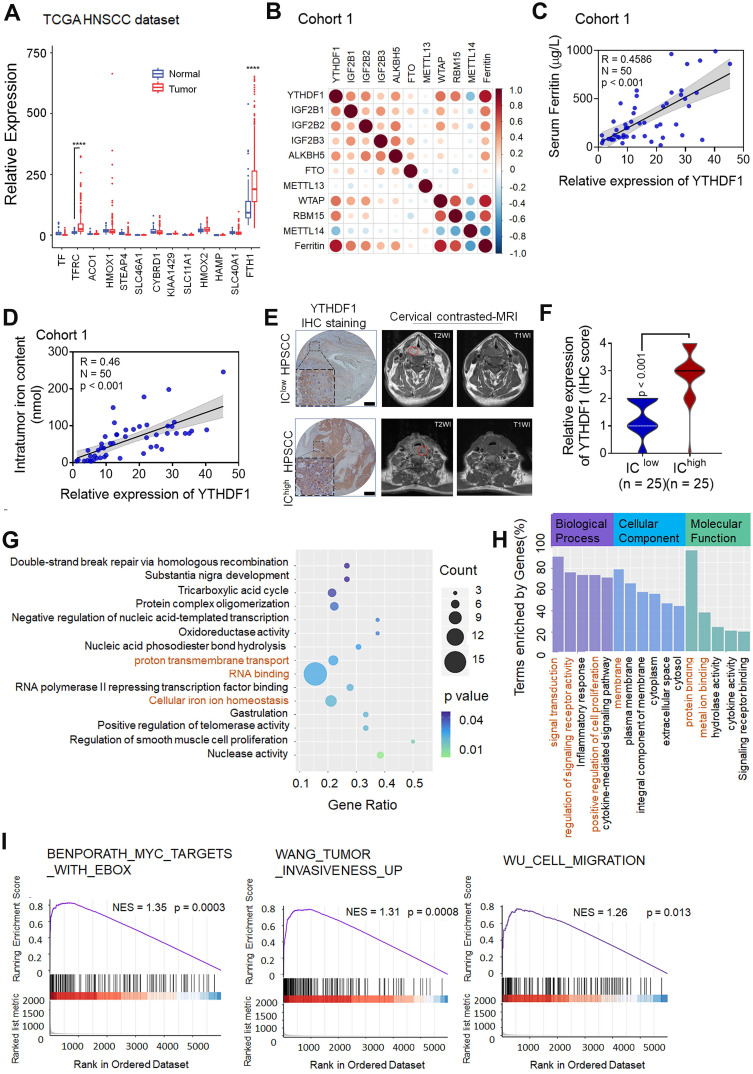
** YTHDF1 is closely correlated with iron metabolism in HPSCC cells. (A)** The results of iron regulatory gene expression assessments determined by RNA-seq with TCGA data are shown. *p < 0.05; **p < 0.01; ***p < 0.001; ***p < 0.0001. **(B)** The correlation matrix shows the relationship between the expression of m6A-modified genes and serum ferritin levels in 50 HPSCC patients in cohort 1. The expression of m6A- modified genes was detected by quantitative real-time PCR (qPCR). **(C)** Correlation between YTHDF1 expression and serum ferritin level in 50 HPSCC patients from cohort 1. YTHDF1 expression was detected by qPCR. **(D)** Correlation between YTHDF1 expression and intratumoral iron content (nmol) in 50 HPSCC patients from cohort 1. YTHDF1 expression was detected by qPCR. **(E)** Representative cervical contrast MR images and YTHDF1 IHC images of samples from HPSCC patients with high and low intratumoral iron concentrations (ICs) in cohort 1. Representative T1- and T2-weighted (WI) cervical MR images of patients with different levels of iron overload. Scale bar = 100 µm (10×, 40×). **(F)** Statistical analysis of the relative expression of YTHDF1 (IHC score) in HPSCC patients with high and low ICs based on unpaired Student's *t*-tests. **(G-H)** Gene Ontology (GO) (G) and Kyoto Encyclopedia of Genes and Genomes (KEGG) (H) analyses of 860 significantly enriched upregulated genes and 889 significantly enriched downregulated genes as identified by RNA-seq. **(I)** GSEA plots showing the pathways of differentially expressed genes altered by YTHDF1 and involved in HPSCC.

To investigate the potential factors and mechanisms involved in intracellular iron levels in the general context of HNSCC, we analyzed the expression levels of iron-related genes by evaluating RNA-sequencing (RNA-seq) data from The Cancer Genome Atlas (TCGA) HNSCC data set, which contains 504 pairs of HNSCC and matched normal specimens. The expression patterns of genes critical for iron metabolism, including aconitase 1 (ACO1, also known as IRP1), cytochrome b reductase 1 (CYBRD1), ferritin heavy chain (FTH1), ferroportin (FPN, also known as SLC40A1), hepcidin antimicrobial peptide (HAMP), SLC11A1 and transferrin receptors (TFRCs), were assessed. Among those iron-related genes, FTH1 and TFRC showed significantly elevated expression **(Figure 1A)**. Next, the expression patterns of m^6^A RNA methylation regulators were assessed, which revealed that the expression levels of multiple regulators were remarkably different between the HNSCC tumor samples and normal control samples **(Figure S1A)**.

Because ferritin is encoded by FTH1 and TFRC is the most important gene for intracellular iron uptake, we concentrated on exploring the correlation between m^6^A modification and both ferritin level and intratumoral iron concentration in HPSCC patients. Analyses of ferritin expression and m^6^A modifications were then undertaken using samples from 50 primary HPSCC patients in **cohort 1** with measured ferritin expression and pre-operative cervical magnetic resonance imaging (MRI). There was a significant positive correlation between serum ferritin and YTHDF1 expression, as measured by serum ferritin concentration **(Figure 1B-C)**. In addition, a significant correlation between intratumoral iron content (nmol) and relative YTHDF1 expression was observed in the HPSCC patients of cohort 1 **(Figure 1D)**. To validate this correlation, intratumoral iron concentration (IC) based on the relaxation rates R2 (1/T2) of the cervical contrast MRI was measured by an experienced radiologist at Sir Run Run Shaw Hospital **(Figure 1E).** The R2 and R2* values generated comparable estimates of non-invasive intratumoral IC in a study by Wood et al 44. Data analysis revealed a remarkable correlation between high IC values and YTHDF1 immunohistochemical (IHC) staining **(Figure 1F)**. However, quantification of RNA methylation revealed that the level of m^6^A modification was not increased in the HPSCC patients with higher serum ferritin, nor did it affect the intratumoral iron content (nmol) **(Figure S1B-C)**.

To elucidate whether YTHDF1 plays a role in HPSCC iron metabolism and tumorigenesis, we performed transcriptome sequencing (RNA-seq) with YTHDF1-knockdown and control HPSCC FaDu cells. YTHDF1 knockdown induced by lentiviral shRNAs was confirmed at both the mRNA and protein levels (**Figure S1D-E**). A total of 34083 genes were identified and quantified by RNA-seq. YTHDF1 knockdown resulted in 1749 significantly altered genes (Student's t-test, *p <* 0.05) comprising 860 upregulated genes (49.1%) and 889(50.8%) downregulated genes **(Figure S1F).** KEGG and GO analysis showed that the following pathways were enriched with these genes: signal transduction, regulation of signaling receptor activity, protein binding, oxidation-reduction process, cellular iron homeostasis and cell proliferation **(Figure 1G-H).** GSEA of the RNA-seq data revealed gene signatures relating to tumor invasiveness, migration and Myc proto-oncogene protein (MYC, a known major contributor to TFRC upregulation in cancer cells), which were enriched in the control HPSCC cells compared with the YTHDF1-knockdown cells, indicating a role for YTHDF1 in HPSCC tumorigenesis and proliferation 45 **(Figure 1I)**. Taken together, these data suggest that YTHDF1 promotes tumor progression and iron metabolism in HPSCC.

### YTHDF1 promotes iron metabolism in HPSCC cells

**Figure 2:**
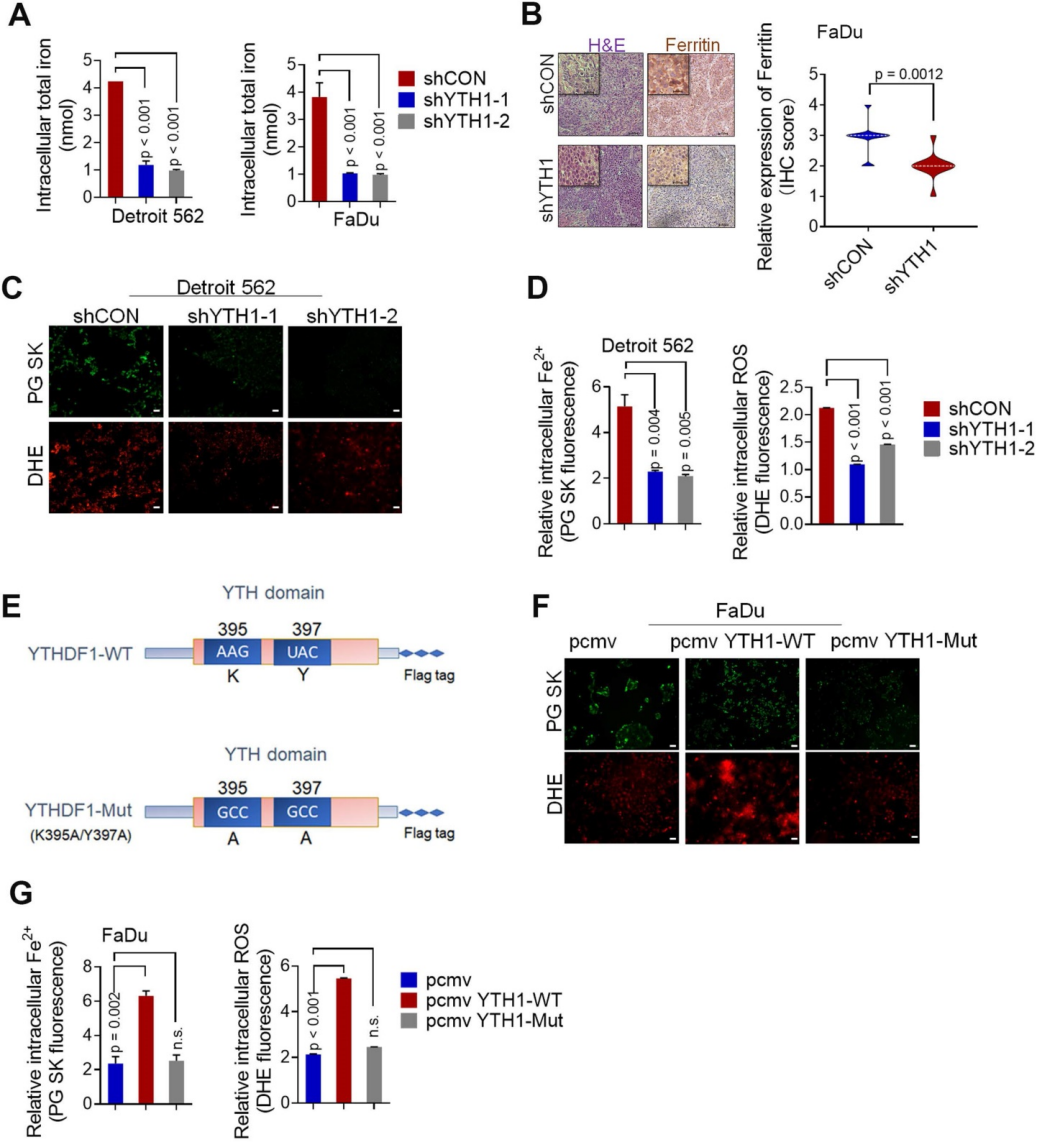
** YTHDF1 promotes iron metabolism in HPSCC cells. (A)** Intracellular iron levels (nmol) were measured using an iron assay kit with Detroit 562 and FaDu HPSCC cells transfected with shCON or shYTHDF1. **(B)** Xenograft tumor masses harvested from shCON- or shYTHDF1-transfected FaDu cells. Representative images of H&E stained cells were used to evaluate ferritin expression. **(C)** Representative fluorescence microscopy was used to evaluate intracellular Fe2+ and ROS levels in Detroit 562 cells transfected with shCON or shYTHDF1 and then stained with Phen Green (green) and LDH (red). Scale bar = 100 µm. **(D)** Quantification of Phen Green- and LDH-positive cells shown in (b), analysed by flow cytometry. The ratio of the mean fluorescence intensity (MFI) was calculated for each sample. The data were normalized to those of the control samples as shown by the relative Fe2+ or ROS ratios. **(E)** Schematic representation of the wild-type (YTHDF1-WT) and mutant (YTHDF1-Mut) YTHDF1 constructs. **(F)** Representative fluorescence microscopy showing intracellular Fe2+ and ROS levels in Detroit 562 cells transfected with a control vector, pCMV-YTHDF1-WT or pCMV-YTHDF1- Mut plasmid and stained with Phen Green (green) and LDH (red) (F). Scale bar = 100 µm. **(G)** Quantification of Phen Green- and LDH-positive cells shown in (e), analysed by flow cytometry. The ratio of the mean fluorescence intensity (MFI) was calculated for each sample. The data were normalized to those of the control samples as shown by the relative Fe2+ or ROS ratios. Means±SEM, unpaired Student's t-tests. WT: Wild-type, YTH1:YTHDF1, PG: Phen Green.

According to the literature, iron accumulation in the cell via extracellular transport into cells or ferritin release result in the formation of a common labile iron (Fe^2+^) pool (LIP) that is available for redox cycling 46,47. The LIP can readily contribute to steady-state levels of ROS in cancer cells through Fe^2+^-dioxygen biochemistry and Fenton reactions, increasing genomic instability and proliferation 48-50. Therefore, the LIP is an active hub in the genesis of cancer that links iron metabolism to the hallmarks of cancer 51. *In vitro* experiments showed that YTHDF1 knockdown significantly decreased total HPSCC intracellular iron levels **(Figure 2A)**, whereas *in vivo* experiments indicated that tumor xenografts with YTHDF1 knockdown had lower ferritin expression, as determined by IHC analysis **(Figure 2B).** To determine whether YTHDF1 directly promotes HPSCC iron metabolism, we conducted intracellular Fe^2+^ and ROS analyses. As predicted, knocking down YTHDF1 remarkably reduced the intracellular Fe^2+^ and ROS levels, as determined by Phen Green SK fluorescence iron staining and dihydroethidium (DHE) ROS staining, respectively **(Figure 2C-D)**. To elucidate whether YTHDF1-regulated iron metabolism is dependent on m^6^A modifications, we generated two point mutations, K395 and Y397, in the YTH domain and added a FLAG tag. Mutating the YTH domain abrogated the binding capacity of YTHDF1 with mRNA **(Figure 2E)**, as reported in previous studies 52,53. Both YTHDF1 wild-type (YTH1-WT) and mutant (YTH1-Mut) recombination plasmids were successfully transfected into FaDu cells **(Figure S1G)**. We found that ectopic expression of YTH1-WT increased intracellular Fe^2+^ and ROS levels, while abrogating the activity of the YTH domain dramatically decreased both intracellular Fe^2+^ and ROS levels in FaDu cells **(Figure 2F-G)**. Considering these results, we suggest that YTHDF1 promotes HPSCC iron metabolism in an m^6^A-dependent manner.

### YTHDF1-induced HPSCC proliferation depends on the promotion of iron metabolism

**Figure 3:**
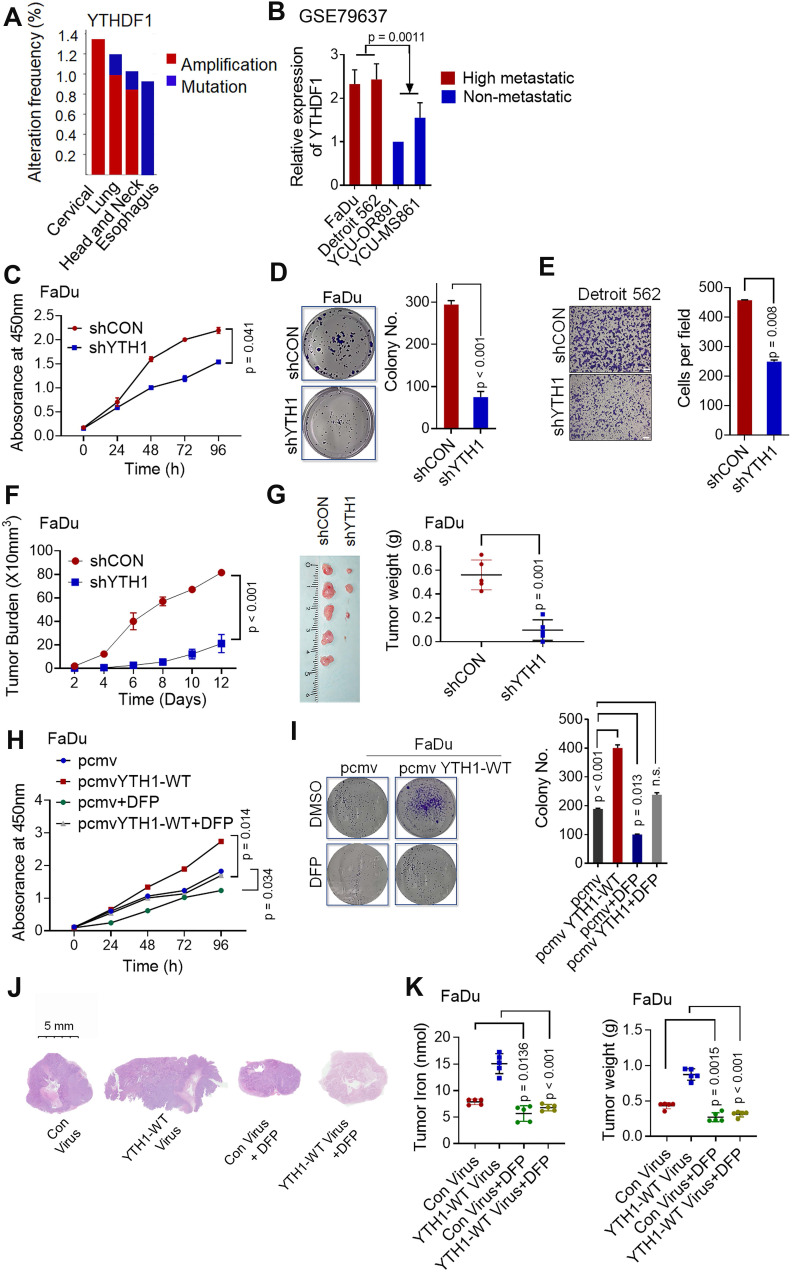
** YTHDF1 promotes HPSCC cell proliferation by regulating intracellular iron metabolism. (A)** YTHDF1 is frequently amplified in various squamous cell cancers (cervical, lung, head and neck, oesophagus, etc.) according to cBioPortal data sets. Colors indicate mutations (green), deletions (blue), and amplifications (red). **(B)** Expression of YTHDF1 mRNA in highly metastatic cell lines (FaDu origin: hypopharynx, Detroit 562 origin: pleural effusion) and nonmetastatic lines (YCU-OR891 origin: oral floor, YCU-MS861 origin: maxillary sinus); data were generated from a network database. **(C-E)** CCK-8 (C), colony formation (D) and Transwell (E) assays were performed to determine the proliferation and growth of HPSCC cells with YTHDF1 knockdown. Magnification: E, 5 ×, scale bar = 100 µm. **(F,G)** Xenograft tumor masses harvested from shCON- and shYTHDF1-transfected FaDu cells. Tumor burden was measured at the indicated time points (F), and tumor weight was measured 12 days after injection (G). **(H,I)** CCK-8 (H) and colony formation (I) assays were performed with FaDu cells transfected with a control vector or pCMV-YTHDF1-WT plasmid and subsequently treated with 1 mM DFP. **(J-L)** Representative images of H&E-stained tissues to evaluate xenograft tumor formation (J), tumor volumes (K), and intratumoral iron levels (L) in nude mice bearing FaDu cells transfected with a control vector or pCMV-YTHDF1-WT plasmid with or without DFP treatment (1 mg/mL in drinking water). The results are presented as the mean± SEM of 5 mice per group per time point, unpaired Student's t-test. WT: wild-type, YTH1: YTHDF1; DFP: deferiprone. **Figure 3K** and **Figure S2E** show the same control virus and YTHDF1-WT virus xenograft groups.

Shown by the cBioPortal network, YTHDF1 is frequently amplified and mutated in various squamous cell carcinomas (cervical, lung, head and neck, and esophageal cancers) **(Figure 3A)**. In addition, based on a GEO dataset (GSE79637) of HNSCC specimens, YTHDF1 expression was more prominent in highly metastatic lines (FaDu origin: hypopharynx, Detroit 562 origin: pleural effusion) than in nonmetastatic lines (YCU-OR891 origin: oral floor, YCU-MS861 origin: maxillary sinus) (**Figure 3B**). The function of YTHDF1 in HPSCC has never been reported. Our functional validation data showed that, compared with the shRNA control cells, Detroit 562 and FaDu cells with YTHDF1 knockdown showed significant reductions in viability **(Figure 3C)**, colony formation **(Figure 3D)**, and migration **(Figure 3E)** *in vitro*. Knocking down YTHDF1 dramatically mitigated tumor growth, as reflected by tumor volumes and weights in the xenograft mouse models **(Figure 3F-G)**. Furthermore, overexpression of YTHDF1-WT increased the proliferation **(Figure S2A)**, colony formation **(Figure S2B)** and migration **(Figure S2C)** of Detroit 562 cells. YTHDF1 overexpression also remarkably increased xenograft tumor volumes **(Figure S2D)** and weights **(Figure S2E)**; however, the same significant oncogenic effect was not observed in cells overexpressing YTHDF1-Mut *in vitro* **(Figure S2A-C)** or *in vivo* **(Figure S2D-E)**. In addition, treatment with deferiprone (DFP, a new generation of intracellular iron chelators in clinical trials 48,54,55) significantly blocked YTHDF1-induced HPSCC iron accumulation, growth and colony formation *in vivo* and *in vitro* **(Figure 3H-L)**. The data strongly suggest that YTHDF1 plays a pivotal role in cancer progression by regulating intracellular iron metabolisms. We also confirmed that the YTHDF1 protein in the xenograft mouse models were not significantly affected upon DFP treatment by Western blot analysis **(Figure S2F)**, excluding the possibility that cellular iron level regulated YTHDF1 levels. These data strongly suggest that YTHDF1 plays a pivotal role in cancer progression by regulating intracellular iron metabolism.

### Transcriptome-wide m^6^A-seq, RNA-seq and RIP-seq assays identify potential targets of YTHDF1 in HPSCC

**Figure 4:**
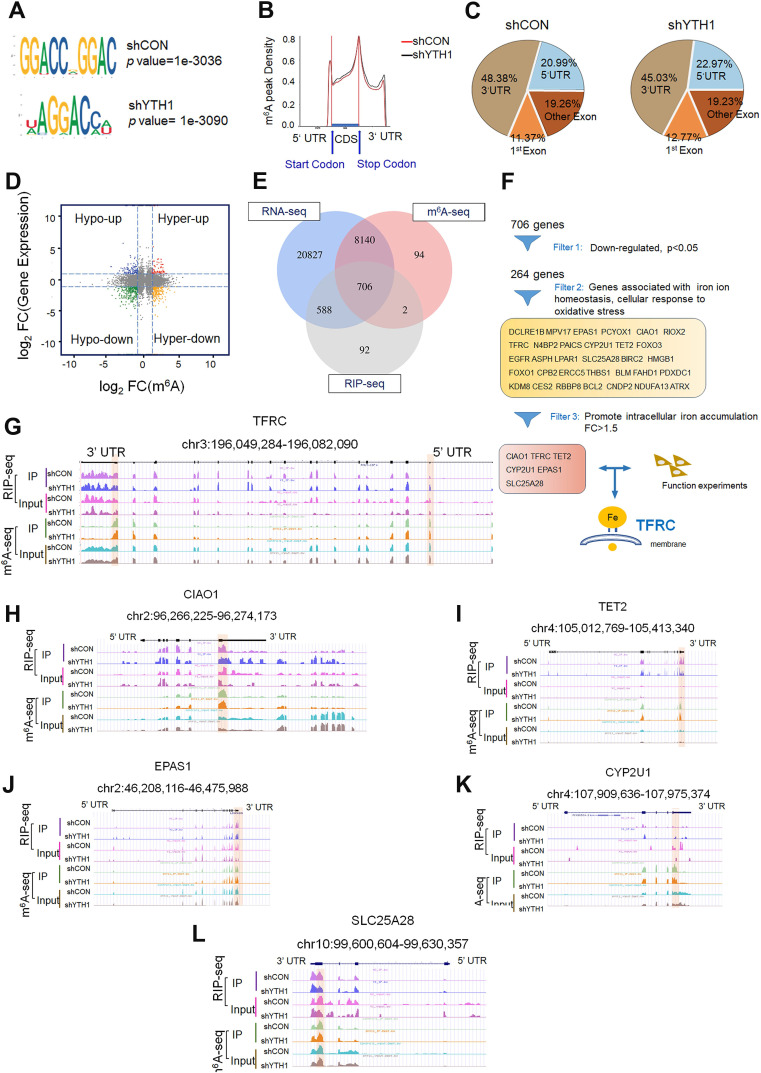
** Transcriptome-wide m^6^A-seq, RNA-seq and RIP-seq assays. (A)** The m^6^A motif detected by the HOMER motif discovery tool with m6A-seq data. Metagene plot depicting nearly unchanged m6A-peak distributions and similar GGAC consensus motifs in the shCON- and shYTHDF1-transfected FaDu cells (both replicates). **(B)** Density distribution of the m^6^A peaks across mRNA transcripts. The upstream untranslated region (5′UTR), coding region (CDS), and downstream untranslated region (3′UTR) were divided into 100 segments, and the percentages of peaks within each segment were determined. **(C)** Proportion of m6A peak distribution in the 5'UTR, start codon, CDS, stop codon and 3'UTR region in the entire set of mRNA transcripts. **(D)** Distribution of genes with a significant change in both m6A level (log2 FC) and gene expression level (log2 FC) in the shCON- and shYTHDF1-transfected FaDu cells. **(E)** Venn diagram illustrating the overlapping genes identified by m6A-seq, RIP-seq, and RNA-seq. **(F)** Flow chart of the selected candidate YTHDF1 target genes in FaDu cells. **(G-I)** IGV tracks displaying the distribution of m^6^A peaks and YTHDF1-binding peaks among the indicated genes according to m^6^A-seq and YTHDF1 RIP-seq of FaDu cells.

To identify and localize m^6^A sites, YTHDF1-knockdown and control FaDu cells were subjected to transcriptome-wide m^6^A-sequencing (m^6^A-seq and MeRIP-seq) assays. Principal component analysis (PCA) showed that two repeats (shCON: control1 and control2; shYTHDF1: shY11 and shY12) of each sample clustered together, suggesting good repeatability among the two replicates of each group **(Figure S3A)**. By applying the HOMER motif discovery tool, we found that the “GGAC” consensus sequence was the primary motif enriched in the m^6^A peaks **(Figure 4A).** In agreement with previous reports, we found that the m^6^A peak density was not significantly changed **(Figure 4B)**. Peaks were located in protein-coding transcripts and enriched in the 5'UTR and 3′UTR, especially near stop codons **(Figure 4B-C)**.

Upon analysis of the RNA-seq data, the general transcription level did not change after YTHDF1 was knocked down in HPSCC cells, suggesting that the loss of YTHDF1 did not change the RNA abundance **(Figure S3B-C)**. A total of 8846 (29.2%) m^6^A-modified transcripts overlapped with the RNA-seq data. The general m^6^A level of transcripts was not significantly changed, as 51.5% (7505 of 14562) of the genes were downregulated, and 48.5% (7050 of 14562) of the genes were upregulated**.** However, the general gene expression of YTHDF1 targets was downregulated in the YTHDF1-knockdown FaDu cells compared with that in the shCON cells **(Figure 4D)**. We verified this result by quantitative analysis of global RNA m^6^A levels in YTHDF1-knockdown and control FaDu cells, as measured with an EpiQuik m^6^A quantification assay. Indeed, no noticeable difference was found in the m^6^A levels of the two samples **(Figure S3D)**. These results were in line with previous findings showing that YTHDF1 does not affect the RNA abundance of its targets but rather regulates protein synthesis by interacting with m^6^A-methylated mRNAs 32,35.

As a crucial m^6^A reader, YTHDF1 promotes the translation of m^6^A-methylated mRNAs and recruits translation initiation factors, thereby significantly improving translation efficiency 56. Therefore, we sequenced RNA obtained from the immunopurified complex of YTHDF1 (RIP-seq) to identify YTHDF1-bound mRNAs. RIP-seq revealed 2450 mRNAs as candidate targets of YTHDF1 **(Figure S3E)**. Overlapping genes found through the RNA-seq, m^6^A-seq, and RIP-seq data analyses showed that 706 genes bound by YTHDF1 were marked with m^6^A **(Figure 4E)**. Among these 706 genes, 264 were downregulated. YTHDF1 is known to bind and affect m^6^A-methylated transcripts 30,57. Therefore, mRNA transcripts of proteins that were downregulated in YTHDF1-knockdown cells were likely potential targets. Moreover, GSEA showed that these 264 downregulated genes are involved in RNA metabolic processes, including GO_RNA binding, Reactome_Metabolism of RNA, GO_cellular iron homeostasis, and GO_Regulation_of_Transmembrane_Transport **(Figure S3F)**. Six genes (CIAO1, TFRC, TET2, CYP2U1, EPAS1, and SLC25A28) with significantly decreased expression in the YTHDF1-knockdown cells (*p* < 0.05, fold change > 1.5) were selected as potential candidates **(Figure 4F)**. Noticeably, the m^6^A peaks fit well with YTHDF1-binding enrichment sequences (m^6^A-seq+RIP-seq) in these transcripts, as shown by Integrative Genomics Viewer (IGV) software **(Figure 4G-L)**. Significant m^6^A peaks as well as YTHDF1 binding enrichment in the TFRC were observed **(Figure 4G)**.

TFRC is overexpressed on the extracellular surface of the plasma membrane in a variety of solid cancer cells, where they enable increased iron uptake 45. Upon release from the TF-TFRC complex into the cytosol, ferric iron is reduced to ferrous iron by ferrireductases within endosomes 58,59. Moreover, TFRC is involved in the gene enrichment of RNA binding, iron homeostasis, and WEI_mycn targets with e-boxes. Several studies have suggested that the group of transcription factors regulating TFRC has oncogenic implications 48. Consistent with our results, previously reported m^6^A, RIP and CLIP data from the m^6^A2Target Database (http://m6a2target.canceromics.org/#/search/TFR) identified TFRC mRNA as a potential target of m^6^A readers (GSE78030, GSE92021, GSE78507, and GSE86214) on the 3'UTR, exons or promoter-TSS sites. In conclusion, TFRC is a functional target of YTHDF1 in HPSCC cells.

### YTHDF1 regulates TFRC expression in HPSCC in an m^6^A methyltransferase- dependent manner

**Figure 5:**
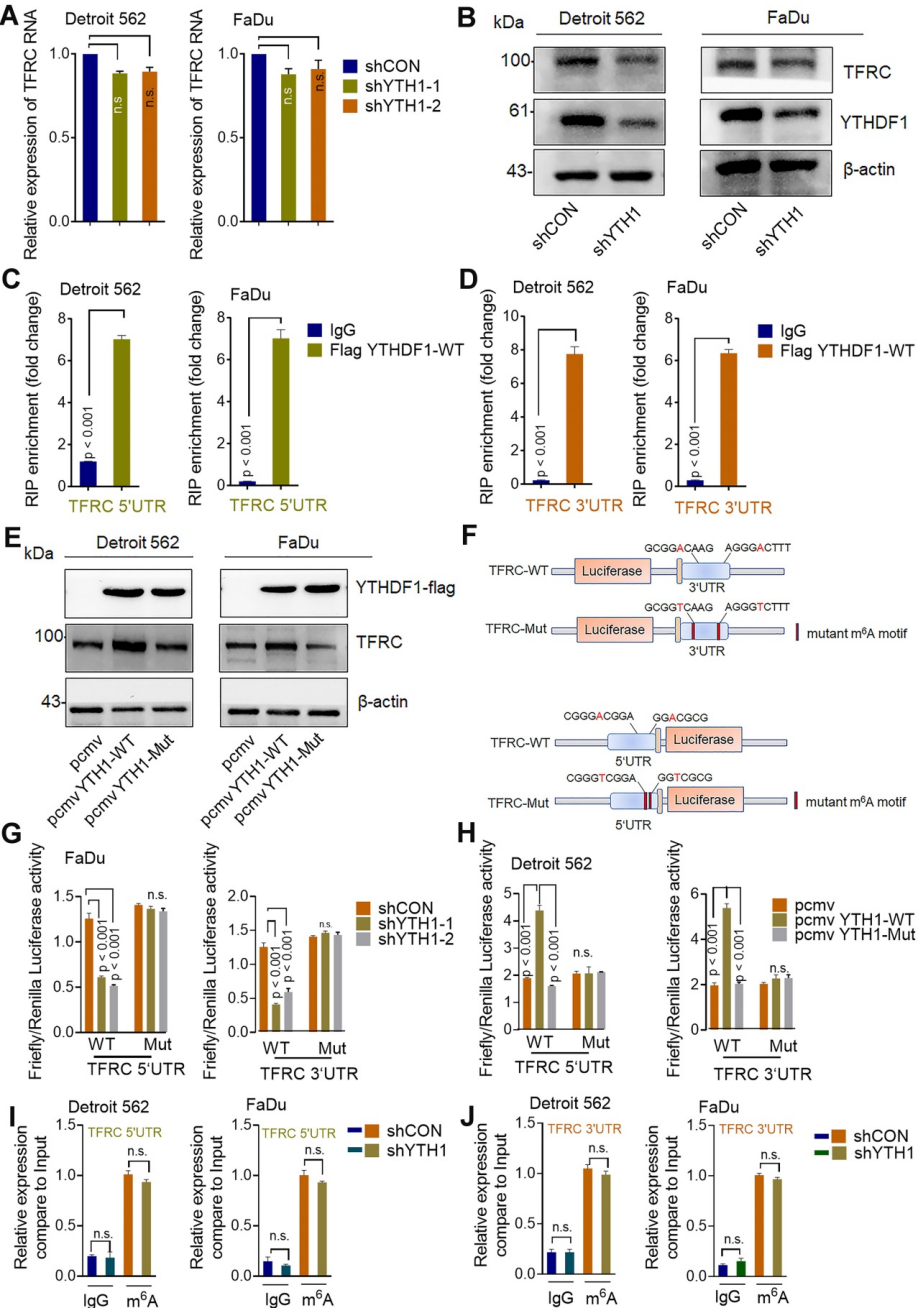
** YTHDF1 regulates TFRC expression in HPSCC cells in an m6A methyltransferase-dependent manner. (A)** Relative RNA level of TFRC in Detroit 562 and FaDu cells upon YTHDF1 knockdown. **(B)** Western blot analysis of the protein level of TFRC in Detroit 562 and FaDu cells upon YTHDF1 knockdown. **(C,D)** RIP analysis of the interaction of the 5'UTR (C) and 3'UTR (D) of TFRC mRNA in FaDu cells transfected with the FLAG-YTHDF1-WT plasmid. Enrichment of TFRC with FLAG was measured by qPCR and normalized to the input level. **(E)** Western blot analysis of the protein level of TFRC in Detroit 562 and FaDu cells transfected with the YTHDF1-WT or YTHDF1-Mut plasmid. **(F)** Schematic representation of wild-type (TFRC-WT) and m6A mutant (TFRC-Mut) TFRC constructs. **(G)** Relative luciferase activity of the WT or Mut TFRC-5′UTR and TFRC-3′UTR luciferase reporter in FaDu cells transfected with control vector or shYTHDF1. Firefly luciferase activity was measured and normalized to Renilla luciferase activity. **(H)** Relative luciferase activity of WT and Mut (A-to-T mutation) TFRC-5′UTR and TFRC- 3′UTR luciferase reporters in Detroit 562 cells transfected with pCMV-YTHDF1-WT or pCMV-YTHDF1-Mut plasmid. Firefly luciferase activity was measured and normalized to Renilla luciferase activity. **(I,J)** The m^6^A modification in the 5'UTR (I) and 3'UTR (J) of TRFC mRNA in Detroit 562 and FaDu cells with YTHDF1 knockdown, as assessed by gene-specific m^6^A-RIP-qPCR assays. Error bars indicate the means ± SEM, n = 3; unpaired Student's t-test.

The best-studied m^6^A-dependent functions of the YTH family include regulation of mRNA stability, translation, splicing and lncRNA-mediated gene silencing 56. YTHDF1 facilitates the translation of m^6^A-modified mRNAs and induces a rapid gene expression response and controlled protein production capacity without changing the overall of mRNA levels 56. To examine whether TFRC is a direct target of YTHDF1, we assessed the transcription and translation of TFRC upon YTHDF1 knockdown. In line with the documented functions of YTHDF1, knocking down YTHDF1 decreased the protein level of TFRC without affecting mRNA expression in both Detroit 562 and FaDu cells **(Figure 5A-B).**

Moreover, RIP using an antibody against FLAG followed by qPCR (RIP-qPCR) revealed that the 3'UTR and 5'UTR of TFRC mRNA were immunoprecipitated effectively in both Detroit 562 and FaDu HPSCC cells transfected with YTHDF1-WT **(Figure 5C-D).** Moreover, we found that cells transduced with YTHDF1-WT but not YTHDF1-Mut exhibited increased protein expression of TFRC **(Figure 5E)**. Additionally, we constructed both WT and mutant TFRC luciferase reporter plasmids, which contained the WT 3'UTR and 5'UTR of TFRC or mutated sequences in which an adenosine residue that undergoes the m^6^A modification was replaced by a T residue (TFRC-m^6^A Mut) **(Figure 5F)**. As expected, compared with shCON cells, shYHDF1 cells showed substantially reduced luciferase activity of the individual reporter constructs carrying the WT 3′UTR and 5'UTR fragments of TFRC, and this decrease was completely abrogated in cells expressing the 3′UTR and 5'UTR mutant variants **(Figure 5G)**. Furthermore, overexpression of WT YTHDF1 but not mutant YTHDF1 significantly increased the luciferase activity of the individual reporter constructs carrying the WT 3′UTR and 5'UTR fragments of TFRC in FaDu cells **(Figure 5H)**. Then, we assessed the m^6^A modification status of TFRC mRNA by a gene-specific m^6^A assay, but no significant enrichment of TFRC mRNA was observed in HPSCC cells **(Figure 5I-J)**. To test whether YTHDF1 contributed to TFRC protein degradation, control and YTHDF1-knockdown Detroit 562 cells were treated with the protein translation inhibitor cycloheximide (CHX) 60,61. Western blot analysis revealed that knocking down YTHDF1 had no effect on the stability of TFRC protein in HPSCC cells **(Figure S4A)**.

Next, we performed the polysome profiling assay. Polysome profiling supported the decreases of 80S monosome assembly and polysomes in shYTHDF1 Detroi562 cells **(Figure S4B)**. The qRT- PCR showed that YTHDF1 knockdown resulted in significant lower TFRC mRNA in translation fractions **(Figure S4C)**. These results support the notion that YTHDF1 regulates protein synthesis while excluding the possibility that YTHDF1 affects TFRC protein stability. Noticeably, TFRC is predominantly regulated by the iron regulatory protein (IRP/IRE) system at both the transcriptional and post-transcriptional levels 62. To determine whether YTHDF1 regulates the IRE/IRP system, we reanalyzed the sequence data. Neither different m^6^A peaks in the mRNA nor significant gene expression of these proteins was observed in the m^6^A-seq data. RIP-seq data indicated that IREB2 and YTHDF1 could bind each other, but RIP-qPCR failed to validate this finding (data not shown). These results confirmed that YTHDF1 enhanced TRFC expression via its methyltransferase domain at translational level.

### TFRC is a crucial target gene for the YTHDF1 promotion of iron metabolism

**Figure 6:**
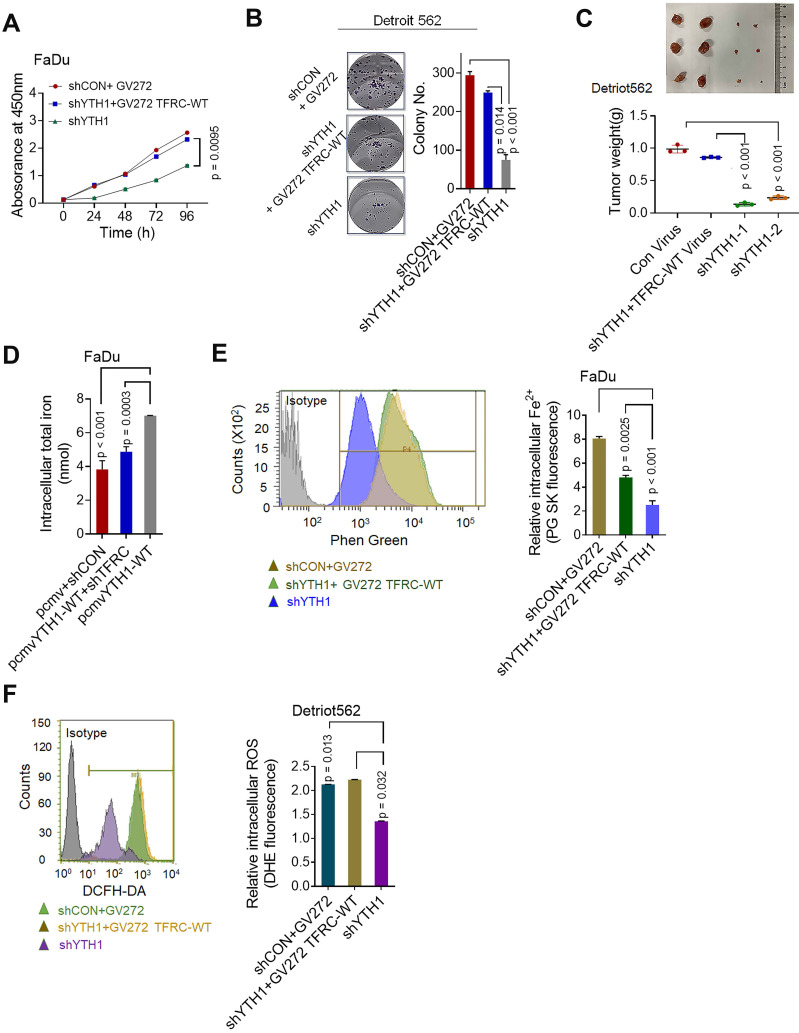
** TFRC is a crucial target gene for the YTHDF1 promotion of iron metabolism. (A,B)** CCK-8 (A) and colony formation (B) assays were performed after shCON and YTHDF1- knockdown Detroit 562 cells were transfected with the GV272 or GV272-TRFC-WT plasmid. **(C)** Masses of the xenograft tumors harvested from different groups. Tumor weight was measured 12 days after injection. **(D)** Intracellular iron levels (nmol) were measured in the GV272- and GV272-TRFC-WT- transfected shCON and YTHDF1-knockdown Detroit 562 and FaDu cells. **(E,F)** Flow cytometry and quantification of Phen Green- and LDH-positive shCON and YTHDF1-knockdown Detroit 562 cells transfected with the GV272 or GV272-TRFC-WT plasmid showing intracellular Fe^2+^ levels (E) and ROS levels (F).

The major mechanism by which m^6^A exerts its effects is determined by m^6^A-binding proteins 56. Therefore, we conducted functional experiments to investigate whether TRFC participates with YTHDF1 in promoting iron metabolism. WT and YTHDF1-knockdown HPSCC cells were transfected with control or TFRC-overexpressing plasmids. The overexpression efficiency of TFRC in Detroit 562 and FaDu cells was confirmed by Western blot analysis **(Figure S5A)**. Ectopic expression of TFRC partially restored the viability **(Figure 6A)**, colony formation ability **(Figure 6B)** and xenograft tumor growth **(Figure 6C)** of YTHDF1-knockdown cells. The efficiency of TFRC shRNAs was confirmed by qPCR and Western blot analysis **(Figure S5B-C)**. TFRC downregulation also led to significantly impaired YTHDF1-induced increases in cell viability and migration **(Figure S5D-E)**. These results strongly indicated that TFRC is a critical target gene of YTHDF1 in HPSCC cells.

The intracellular iron level and ROS were synchronously assessed. As expected, TRFC overexpression restored the reductions in intracellular iron content, Fe^2+^ levels and ROS levels in YTHDF1-knockdown cells **(Figure 6E-F).** Furthermore, the downregulation of TFRC significantly reduced YTHDF1-mediated increases in iron content, intracellular Fe^2+^ level and ROS level in FaDu cells **(Figure S5F-H)**. As TFRC expression led to increased iron uptake, we next measured the expression of STEAP4, an important ferrireductase that facilitates increased iron uptake by cells 63. FTH1 is a protein with ferroxidase activity that facilitates the conversion of excess Fe^2+^ to Fe^3+^ and then stores it in ferritin. Western blot analysis showed that TFRC downregulation also reduced the YTHDF1-induced increase in the protein levels of STEAP4 and FTH1, TRFC overexpression restored the protein levels of STEAP4 and FTH1 in YTHDF1-knockdown cells **(Figure S5I)**. Taken together, the data show that TRFC mediates the iron regulatory function of YTHDF1 in HPSCC cells.

### YTHDF1 is linked to poor prognoses of HPSCC patients receiving CCT/RT treatment

**Figure 7:**
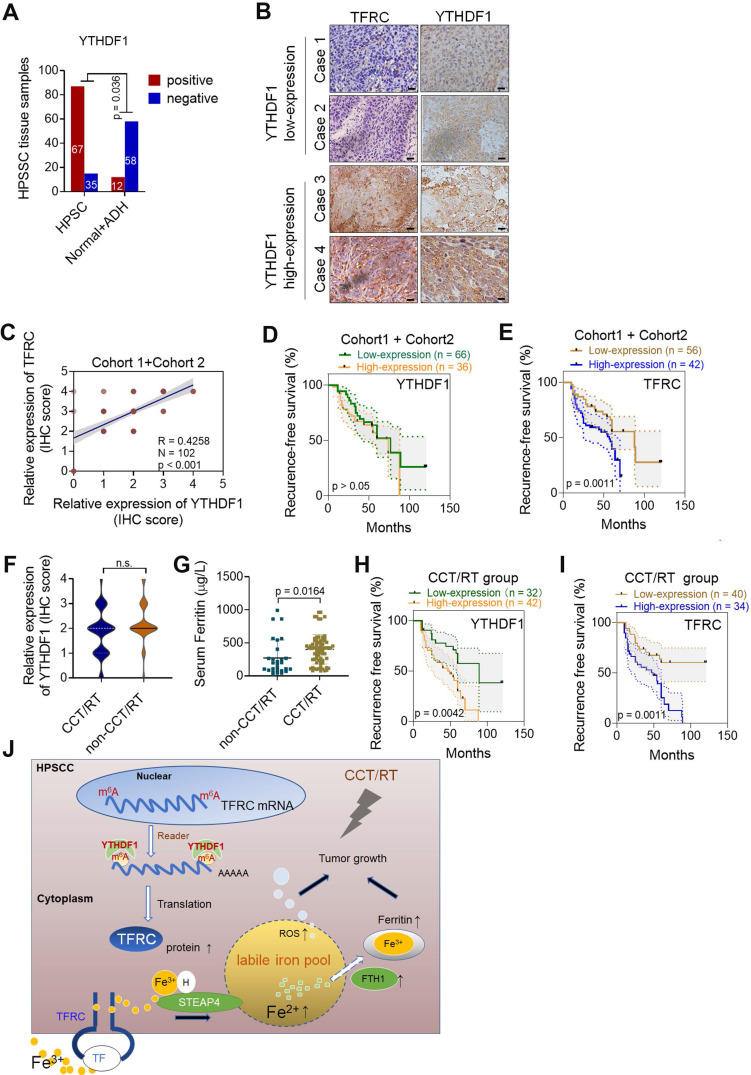
** YTHDF1 links poor prognosis in HPSCC patients with CCT/RT treatments. (A)** Quantification of YTHDF1 expression in cancerous and paired paracancerous tissues from patients in cohort 1 and cohort 2. **(B)** Representative IHC images of YTHDF and TFRC in HPSCC tissues from patients in cohort 1 and cohort 2. Scale bar = 100 µm (40 ×). **(C)** Pearson's rank correlation of YTHDF1 and TFRC proteins in HPSCC tissues from patients in cohort 1 and cohort 2 based on the IHC analysis. **(D,E)** Kaplan-Meier analysis of HPSCC patients to determine the correlations between YTHDF1 expression (D), TRFC expression (E) and recurrence-free survival based on data generated from IHC staining of HPSCC tissues. **(F,G)** Statistical analysis of the relative expression of TFRC (F) and serum ferritin (G) in HPSCC patients treated with or without CCT/RT, as assessed by the Mann-Whitney U test. **(H,I)** Kaplan-Meier analysis of HPSCC patients treated with CCT/RT to determine the correlations between YTHDF1 expression (H), TRFC expression (I) and recurrence-free survival. **(J)** Proposed model of the relationship between TFRC expression enhanced by the m^6^A modification reader YTHDF1, HPSCC cell progression, cell response to CCT/RT upon tumor hypoxia, and iron metabolism.

We next performed IHC analyses of HPSCC and paracancerous tissues from patients in **cohort 1** and **cohort 2**. The percentage of YTHDF1-expressing cells was significantly higher in HPSCC tissues (65.7%; 67/102) than in normal epithelium tissues (17.1%; 12/70) **(Figure 7A)**. A significant difference was found between the clusters of T stage and N stage tissues **(Figure 6SA-B).** In addition, samples with higher YTHDF1 expression showed intense TFRC staining, while samples with low YTHDF1 expression displayed lower levels of TFRC staining **(Figure 7B-C).** Surprisingly, Kaplan-Meier analysis showed that elevated TFRC expression in HPSCC cells was associated with poor prognosis; however, patients with high YTHDF1 expression did not have shorter disease-free survival, in contrast to our expectations **(Figure 7D-E)**. We obtained similar results when analyzing the TCGA head and neck dataset **(Figure 6SC-D).**

Then, we reanalyzed the clinical characteristics and treatments for patients in **cohort 1** and **cohort 2.** We found that 72.5% of the patients were treated with postoperative platinum-based chemoradiotherapy (CCT) or radiation (RT) alone. High TFRC expression correlated with a reduced response to chemoradiotherapy because it enhanced iron uptake and storage 10,64. Based on the significant increase in intratumoral IC and serum ferritin levels in HPSCC tissues, we suspected that YTHDF1 might also ameliorate the response to radiation because of TFRC-induced iron accumulation. Then, we reassessed the association between YTHDF1 staining and recurrence-free survival in patients treated with adjuvant CCT/RT. As expected, YTHDF1 did not differ between patients who receives CCT/RT treatment and those who did not **(Figure 6SE)**, but TFRC expression and serum ferritin levels were significantly upregulated in patients who received adjuvant CCT/RT treatment **(Figure 7F-G).** High YTHDF1 and TFRC levels were associated with poor prognosis in patients receiving adjuvant CCT/RT treatments **(Figure 7H-I).** Taken together, these data suggest that YTHDF1 enhances TFRC expression in HPSCC through an m^6^A- dependent mechanism.

## Discussion

Although the m^6^A modification has been researched for decades, the potential involvement of m^6^A modification on RNA is poorly defined in the context of human HNSCC and iron metabolism. Data from epidemiological studies and *in vivo* and *in vitro* models have corroborated the critical role of iron in HNSCC 21,22,65,66. Because of its high invasive capacity at all subsites, HPSCC was the focus of our study. Here, through bioinformatics analysis, sample detection, transcriptome-wide high-resolution m^6^A-seq, RNA-seq and RIP-seq assay analyses, and cell biological analyses, we demonstrated that the m^6^A reader YTHDF1 regulates HPSCC iron uptake via m^6^A-modified TFRC mRNA, thereby regulating tumorigenesis and tumor proliferation. Moreover, high expression of YTHDF1 might also be linked to radiation resistance by promoting intratumoral iron accumulation **(Figure 7J)**. Through bioinformatics analyses, we demonstrated that the expression of iron-related genes, especially FTH1 and TFRC (which regulate ferritin and the intratumoral iron levels, respectively), were elevated in cancer tissues. Noticeably, among the m^6^A readers, YTHDF1 had remarkably different expression levels between HNSCC tumor samples and normal control samples. Both upregulated transcription and amplified DNA copy number of YTHDF1 have been observed in various human cancers, making this protein a robust and reproducible cancer biomarker 37. YTHDF1 was also found to maintain intestinal stem cell features during Wnt-driven intestinal regeneration and tumorigenesis 37. Our findings supported the notion that YTHDF1 is the major effector of m^6^A during tumorigenesis. In addition, we firstly demonstrated the relationship between iron metabolism and YTHDF1.

Despite exhibiting generally comparable m^6^A RNA methylation levels, HPSCC patients with higher serum ferritin and intratumoral iron levels show highly upregulated m^6^A reader YTHDF1 expression compared to those patients with lower serum ferritin and intratumoral iron. Combining the results from GO, KEGG and GSEA demonstrated that cellular iron homeostasis and cell proliferation were significantly enriched in response to YTHDF1 knockdown in HPSCC cells. *In vitro* and *in vivo* experiments indicated that downregulating YTHDF1 suppressed cancer growth, colony formation, and immigration and reduced intracellular iron content, Fe^2+^ and ROS levels in HPSCC cells. Importantly, YTHDF1 mutant plays dominant-negative effect to inhibit the function. The current data indicate that m6A can be recognized by proteins that contain a YTH (YT521B homology) domain 56. Therefore, YTH domain functional deletion would interrupt the binding of YTHDF1 to its m6A- methylated targets in our study, deletion of the YTH domain of YTHDF1 blocked YTHDF1-induced iron metabolism in HPSCC cells. Overexpression YTHDF1 using YTHDF1-WT virus xenograft mouse models increased the tumorigenesis *in vivo* **(Figure 3H-L)**. But overexpression YTHDF1-mut did not have such effect **(Figure S2D-E)**. In addition, DFP (intracellular iron chelators) treatment significantly blocked YTHDF1-induced iron uptake and tumorigenesis *in vivo* **(Figure 3H-L)**. These results elucidated that YTHDF1-induced HPSCC iron metabolism depends on its RNA m^6^A function, consistently with other data showing that during tumor development, YTHDF1 activates the translation of methylated mRNAs for sufficient protein production 67. A recent study by Shi, Y et al investigated the function of YTHDF1 in non-small-cell lung cancer (NSCLC) under hypoxia 31. Downregulated YTHDF1 in NSCLC has a protective role against cellular stresses, such as hypoxia and DDP treatment, through the Keap-Nrf2 axis. We found that YTHDF1 increased ROS levels upon regulating iron metabolism, expanding the function of YTHDF1 in intracellular ROS regulation.

YTHDF1 was only once reported to bind to the m6A site in CDS region of Snail mRNA and enhance its translation by recruiting the translation elongation factor eEF-2. They also firstly assessed the most common sites 3'UTR, but they found that it was not involved in m^6^A modification regulated target gene expression. They speculated that YTHDF1 could function via regulating translation elongation as well as translation initiation. Here in our study, m^6^A methylation on 5'UTR and 3'UTR are the main locations of m^6^A modification. YTHDF1 increases the expression of the TFRC gene mainly at the 3′UTR and 5′UTR, which in turn leads to the upregulation of TFRC at the protein level but not at the mRNA level. Notably, mutagenesis assays indicate that the m^6^A sites in the 5'UTR and 3'UTR of TFRC mRNA are essential for YTHDF1 to post-transcriptionally regulate TRFC expression. This finding was further supported by RIP-qPCR and Western blot data. Polysome profiling assay supported that YTHDF1 regulates TFRC translations efficiency. mRNA transcripts with m^6^A modifications have differing fates primarily based on their interaction with different m^6^A readers, which contain a YTH domain, IGF2BPs or eukaryotic initiation factor 3 (eIF3) 56. YTHDF1 did not have a substantial effect on mRNA stability, as indicated by an analysis of DF1-depleted cells 30. In contrast to YTHDF1, YTHDF2, YTHDF3, and YTHDC2 tend to reduce gene expression by promoting m^6^A-modified mRNA decay, while IGF2BPs regulate gene expression by promoting mRNA stability 68. In this study, we did not observe a change in m^6^A modification in TFRC mRNA upon alterations in YTHDF1 expression. In addition, the protein level but not the mRNA level of TFRC declined upon YTHDF1 knockdown. Importantly, our polysome profiling assay showed that YTHDF1 knockdown resulted in significant lower TFRC mRNA in translation fractions, supporting the notion that YTHDF1 regulates protein synthesis.

Whether YTHDF1 interacts with other m^6^A writers and erasers in HPSCC cells remains unknown and needs further investigation. We speculated that YTHDF1 enhanced TFRC expression by promoting translation, which was revealed by an analysis of previously reported transcriptome- wide m^6^A, RIP and CLIP data (GSE78030, GSE92021, GSE78507, and GSE86214). YTHDF1 was shown to promote translation elongation and translation initiation in multiple studies 32,35,53,69. Further studies should focus on initiation factor identification and determine how YTHDF1 promotes translation upon the binding of these initiation factors 56.

We found that high YTHDF1 and TFRC levels were associated with poor prognosis in patients treated with CCT/RT. Interestingly, increased ferritin concentrations were also observed in this group. A previous study demonstrated that cancer cells preferentially exhibit elevated expression of TFRC, which can increase iron uptake 48. Logically, by regulating TRFC-induced iron uptake, YTHDF1 is expected to cause CCT/RT resistance in HPSCC, thereby causing poor prognosis. Thus far, we have shown that YTHDF1 may function as an oncogene by enhancing TFRC protein expression in HPSCC cells, which results in increased iron uptake. Ultimately, imported iron enters the bioactive LIP for proliferation and metabolic purposes 10. However, we cannot definitively point to a direct link between YTHDF1 and LIP. The amount of LIP is detected by post-transcriptional mechanisms of IRP1 and IRP2 62. In our pilot experiments, we failed to observe binding between YTHDF1 and IRP1/IRP2 in our culture systems (data not shown). However, we hypothesized that TFRC is a crucial target gene for YTHDF1-mediated promotion of iron metabolism in HPSCC. Previous studies have demonstrated that TRFC-induced iron uptake is the most important mechanism by which cancer cells internalize iron 70,71. Here, we first revealed that YTHDF1 enhanced TRFC expression via m^6^A recognition to promote iron metabolism. Multiple strategies for anticancer therapies, including utilization of TFRC-mediated cytotoxic drug conjugates and iron chelators, have been designed to disrupt the intracellular iron balance needed to meet the urgent metabolic demand of cancer cells 62. In our study, DFP treatment significantly blocked YTHDF1- induced HPSCC growth and colony formation *in vitro* and *in vivo*. Fe^2+^ is an essential cofactor for the delivery of oxygen to cells, protecting cancer cells from hypoxia-induced stress. However, excessive intracellular iron accumulation can cause cells to experience extreme oxidative stress and may induce tumor death. Iron-dependent cancer death, also known as “ferroptosis”, can be inhibited by iron chelators. The functions and mechanisms of TFRC in cancer cell ferroptosis have been observed, but their relevance is still unclear 72. In other words, TFRC regulation may mark a watershed event during iron accumulation. Depending on primary recognition by the various m^6^A readers, mRNA transcripts with m^6^A modifications undergo different fates 56. Therefore, understanding the functional mechanism of YTHDF1 may enable the reconstitution of TFRC activity, thus enabling the development of specific therapeutic targets according to different intratumoral iron levels.

## Conclusions

In summary, we found that YTHDF1 is closely correlated with iron metabolism and tumor progression in HPSCC. Mechanistically, TFRC was identified as a direct target of YTHDF1 in HPSCC cells and promoted iron metabolism, thereby increasing tumor growth and proliferation. From a therapeutic perspective, targeting YTHDF1 and TFRC-mediated iron metabolism may be a promising strategy for HPSCC.

## Supplementary Material

Supplementary figures and tables.

## Abbreviations

ADH: atypical hyperplasia
ALOX15: arachidonate 15-lipoxygenase
CHX: cycloheximide
CP: ceruloplasmin
CYP4F11: cytochrome P450 family 4 subfamily F member
DFP: deferiprone DFO deferoxamine
EGLN3: Egl-9 family hypoxia inducible factor
FTH1: ferritin heavy chain 1
FTL: ferritin light chain
FTR1: ferroportin 1
GTEx: genotype-tissue expression
HNSCC: head and neck squamous cell carcinoma
HPSCC: hypopharyngeal carcinomas
IC: iron concentration
IHC: immunohistochemical staining
KEGG: Kyoto Encyclopedia of Genes and Genomes
LCN2: lipocalin 2
MRI: magnetic resonance imaging
MYC: Myc proto-oncogene protein
m^6^A: N6-methyladenosine
NSCLC: non-small cell lung cancer
ROS: reactive oxygen species
STEAP4: six transmembrane epithelial antigen of prostate
TFRC: transferrin receptors
YTHDF: YTH domain-containing family
