# Supplementary material for: YTHDF1-enhanced iron metabolism depends on TFRC m6A methylation
Source: Theranostics. 2020 Oct 26;10(26):12072–89. doi: 10.7150/thno.51231 (PMC7667694; doi:10.7150/thno.51231)
Supplement: Supplementary file 1 — Supplementary figures and tables. [file thnov10p12072s1.zip › supplementary materials/supplementary data.pdf]

Supplementary materials

Contents

Figure S1 3
Figure S2 4
Figure S3 6
Figure S4 8
Figure S5 10
Figure S6 11
Supplementary data 1 :HPSCC patients in cohort 1 and cohort 2 11
Supplementary data 2 : Original M6A peak, RIP data, overlapping M6A peaks and RIP data. xlsx 12
Supplementary data 3: Primer list and mutation sequences. Xlsx 13
Supplementary data 4: Supplementary Materials and Methods 14
Patient specimens 14
Global m6A measurement 14
Transcriptome-Seq and M6A-seq 14
Anti-m6A immunoprecipitation and RNA immunoprecipitation(RIP) 15
Constructs and cell culture 15
Lentivirus and Plasmid construction 15
Lentivirus packaging and generation of stable cell lines 15
Cell growth, cell proliferation, migration 15
Iron assay, Intracellular chelatable iron (Fe^{2+}) and ROS measurement 16
Histology, immunohistochemical staining and immunofluorescence staining 16
Total RNA extraction and quantitative real-time PCR(qPCR) 16
Western Blot Analysis 16
Mouse treatment and tumor biology studies 17
Polysome profiling. 17
Luciferase assay 17
Cellular Labile Iron Pool Quantification 17
Protein stability 18
Statistics methods 18
Reference: 18
Supplementary data 5: R code used for data analysis 19

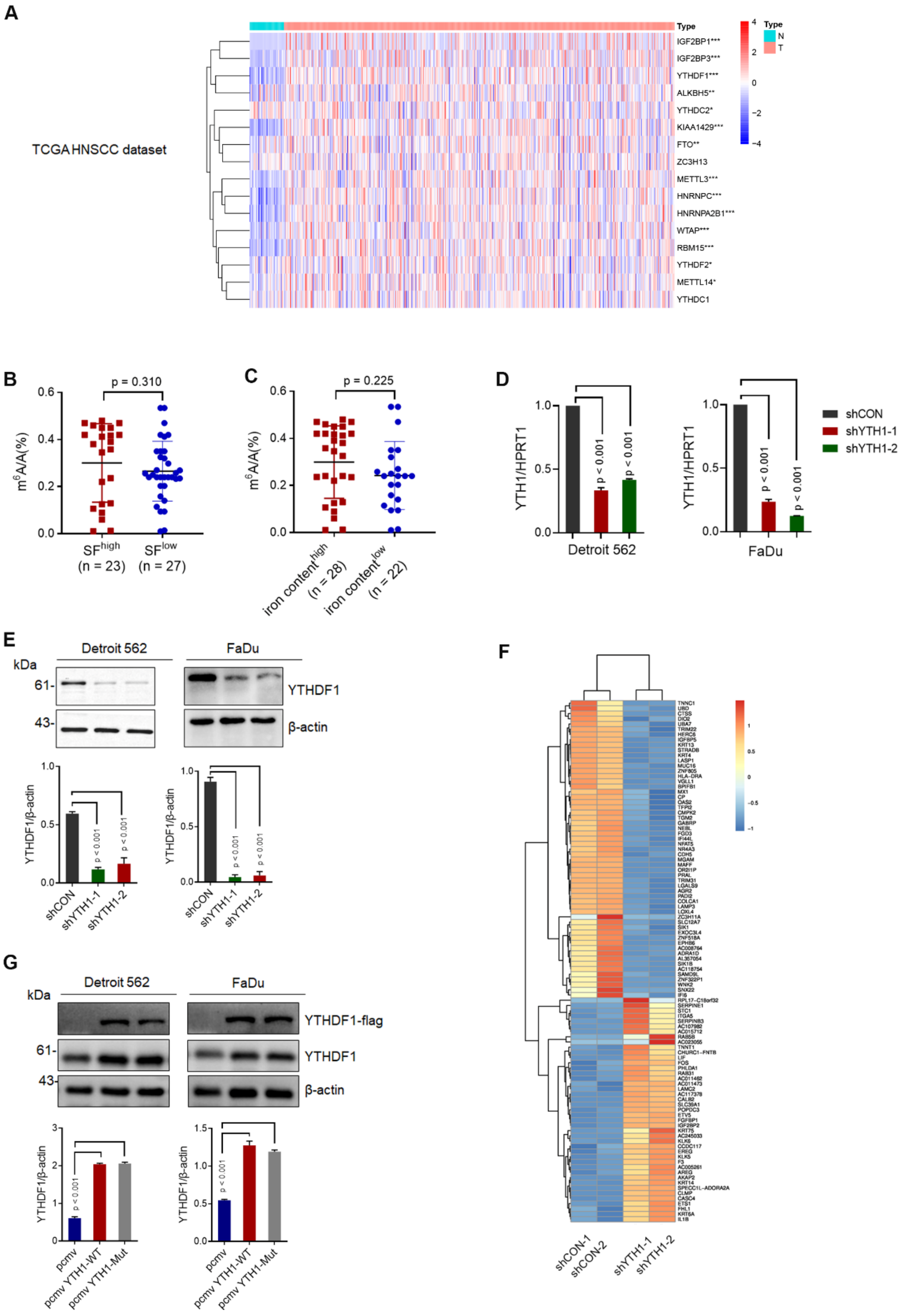
A
TCGA HNSCC dataset
Type
IGF2BP1***
IGF2BP3***
YTHDF1***
ALKBH5**
YTHDC2*
KIAA1429***
FTO**
ZC3H13
METTL3***
HNRNPC***
HNRNPA2B1***
WTAP***
RBM15***
YTHDF2*
METTL14*
YTHDC1
Type
N
T
B
p = 0.310
m6A/A(%)
SFhigh (n = 23)
SFlow (n = 27)
C
p = 0.225
iron contenthigh (n = 28)
iron contentlow (n = 22)
D
YTH1/HPRT1
p < 0.001
Detroit 562
FaDu
shCON
shYTH1-1
shYTH1-2
E
Detroit 562
FaDu
kDa
61-
43-
YTHDF1
β-actin
YTHDF1/β-actin
shCON
shYTH1-1
shYTH1-2
F
shCON-1
shCON-2
shYTH1-1
shYTH1-2
G
Detroit 562
FaDu
kDa
YTHDF1-flag
61-
YTHDF1
43-
β-actin
YTHDF1/β-actin
p < 0.001
pcmv
pcmv YTH1-WT
pcmv YTH1-Mut

Figure S1

A. Expression levels of m^6A RNA methylation regulators in tumor samples and normal control samples from the TCGA HNSCC database are shown by a heatmap.

B. Quantification of m^6A levels in HPSCC patients from cohort 1 with low and high serum ferritin levels. Nonparametric Mann-Whitney U test.

C. Quantification of m^6A levels in HPSCC patients from cohort 1 with low and high intratumoral iron content (nmol). Nonparametric Mann-Whitney test.

D, E. Validated efficiency of shRNAs targeting YTHDF1 as indicated by both real-time RT-PCR (D) and Western blot analysis (E) of Detroit 562 and FaDu HPSCC cells.

F. Heat map of the 1749 most significantly altered proteins based on their intensities and hierarchical clustering analysis; 860 genes were upregulated genes, and 889 genes were downregulated genes as identified by RNA-seq (two replicates).

G. Western blot analysis confirmed YTHDF1 expression in Detroit 562 and FaDu cells cotransfected with empty vector, WT YTHDF plasmid or mutant YTHDF1 plasmid.

N: normal tissue T: cancerous tissue SF: serum ferritin

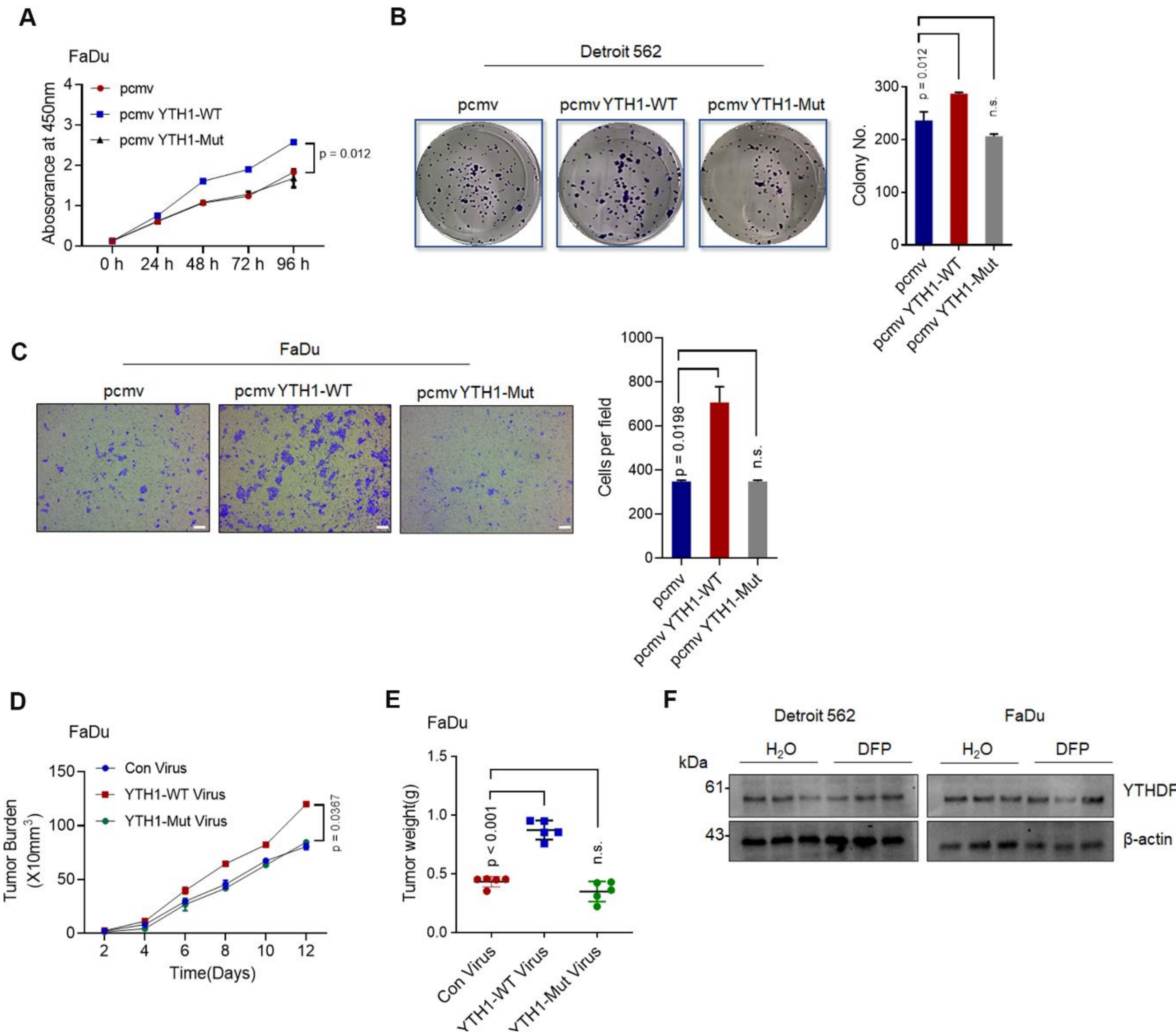

Figure S2

A, B, C. CCK-8 (A), colony formation (B) and migration (C) assays were performed with FaDu cells transfected with a control vector, pCMV-YTHDF1-WT or pCMV-YTHDF1-Mut.

D, E. Xenograft tumor masses harvested from control virus, YTHDF1-WT virus or YTHDF1-Mut virus FaDu xenograft mouse model. Tumor burden was measured at the indicated time points (D), and tumor weight was measured 12 days after injection (E). The results are presented as the mean ± SEM of 5 mice per group per time point.

F. Western blot analysis of TFRC protein expression upon DFP treatment. For DFP treatment, male nude mice at 4 weeks of age were injected subcutaneously into the right flank with Detroit 562

and FaDu cells. cell lines (1 × 10^6 cells). After 12 days, mice were divided into control (H_2O) and DFP groups (n = 5 per group). DFP concentration: 1 mg/mL in the drinking water in for 12 days. Xenograft tumor masses were harvested at Day 24.

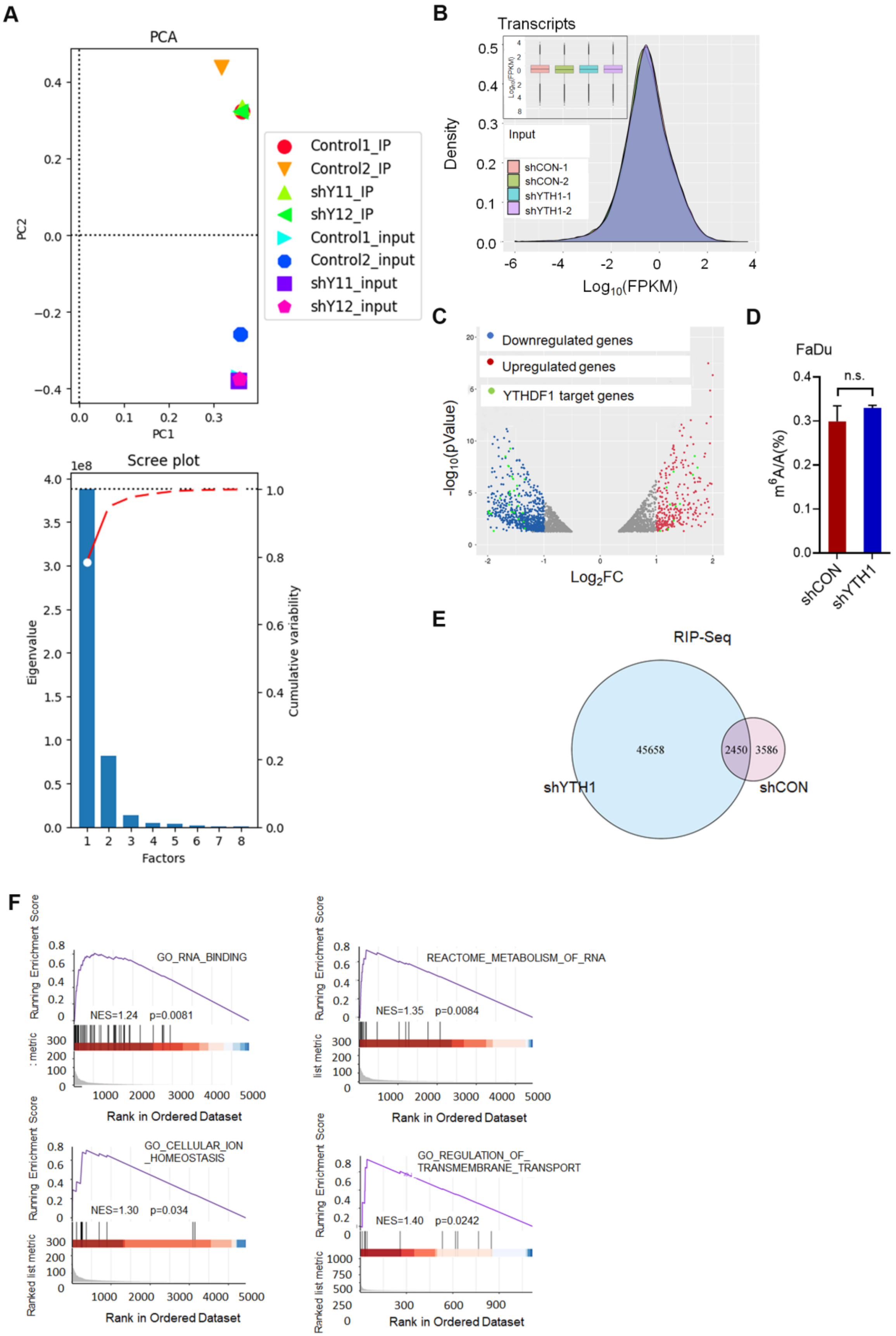
A
PCA
PC1
PC2
Control1_IP
Control2_IP
shY11_IP
shY12_IP
Control1_input
Control2_input
shY11_input
shY12_input
Scree plot
1e8
Eigenvalue
Cumulative variability
Factors
B
Transcripts
Density
Input
shCON-1
shCON-2
shYTH1-1
shYTH1-2
Log10(FPKM)
C
Downregulated genes
Upregulated genes
YTHDF1 target genes
-log10(pValue)
Log2FC
D
FaDu
n.s.
m6A/A(%)
shCON
shYTH1
E
RIP-Seq
45658
2450
3586
shYTH1
shCON
F
Running Enrichment Score
GO_RNA_BINDING
NES=1.24 p=0.0081
Rank in Ordered Dataset
REACTOME_METABOLISM_OF_RNA
NES=1.35 p=0.0084
GO_CELLULAR_ION
_HOMEOSTASIS
NES=1.30 p=0.034
GO_REGULATION_OF_
TRANSMEMBRANE_TRANSPORT
NES=1.40 p=0.0242
Ranked list metric

Figure S3

A. Principal component analysis (PCA) showing that two repeats (shCON: control1 and control2, shYTH1: shY11 and shY12) of each sample clustered together.

B. Cumulative curves and box plot showing transcript expression levels (log_{10}FPKM) in the shCON- and shYTHDF1-transfected FaDu cells (both replicates).

C. Volcano plots of genes with differential expression in shCON- and shYTHDF1-expressing cells (both replicates) (adjusted $p \leq 0.05$ and fold change ≥ 2 or ≤ 0.5). YTHDF1 m^6A target genes are shown as green circles. Two-sided P values were calculated by the likelihood ratio test and adjusted by the Benjamini–Hochberg method; n = 4 (2 conditions × 2 biological replicates).

D. m^6A quantification of shCON- and shYTHDF1-expressing FaDu cells, unpaired Student's t-test.

E. Identification of 2450 YTHDF1-bound mRNAs as revealed by RIP-seq.

F. GSEA plots showing the pathways of the overlapping genes identified by m^6A-seq, RIP- seq, and RNA-seq.

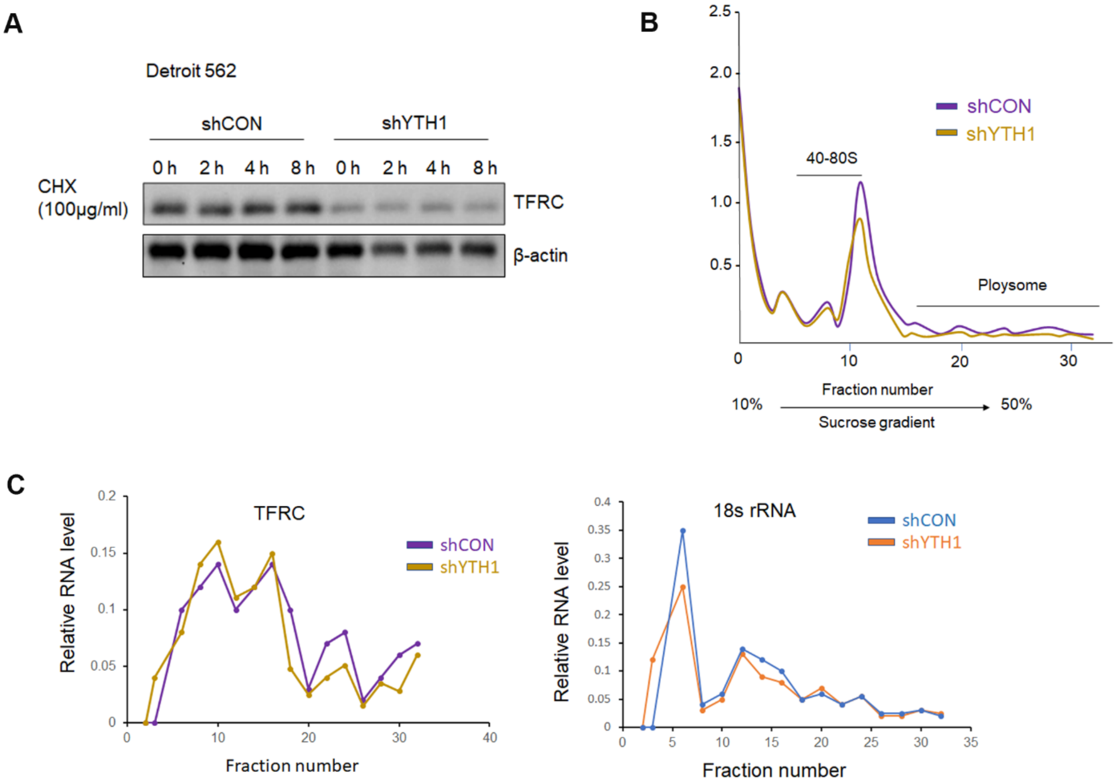

Figure S4

A. Western blot analysis of TFRC protein expression in Detroit 562 cells treated with CHX.

B. RNA fractions were separated as: non-translating fraction (< 40 S), translation initiation fraction (including 40S ribosomes, 60S ribosomes, 80S monosomes, and < 80S) as well as translation active polysomes (> 80S) from shCON and shYTHDF1 Detroit 562 cells. The absorbance at 260 nm was measured during fractionation of polysomes from Detroit 562 cells transiently transfected with shCON or shYTHDF1.

C. The abundance of TFRC transcripts was measured by qPCR in each fraction from the polysome profiling, with 18S rRNA as a negative control.

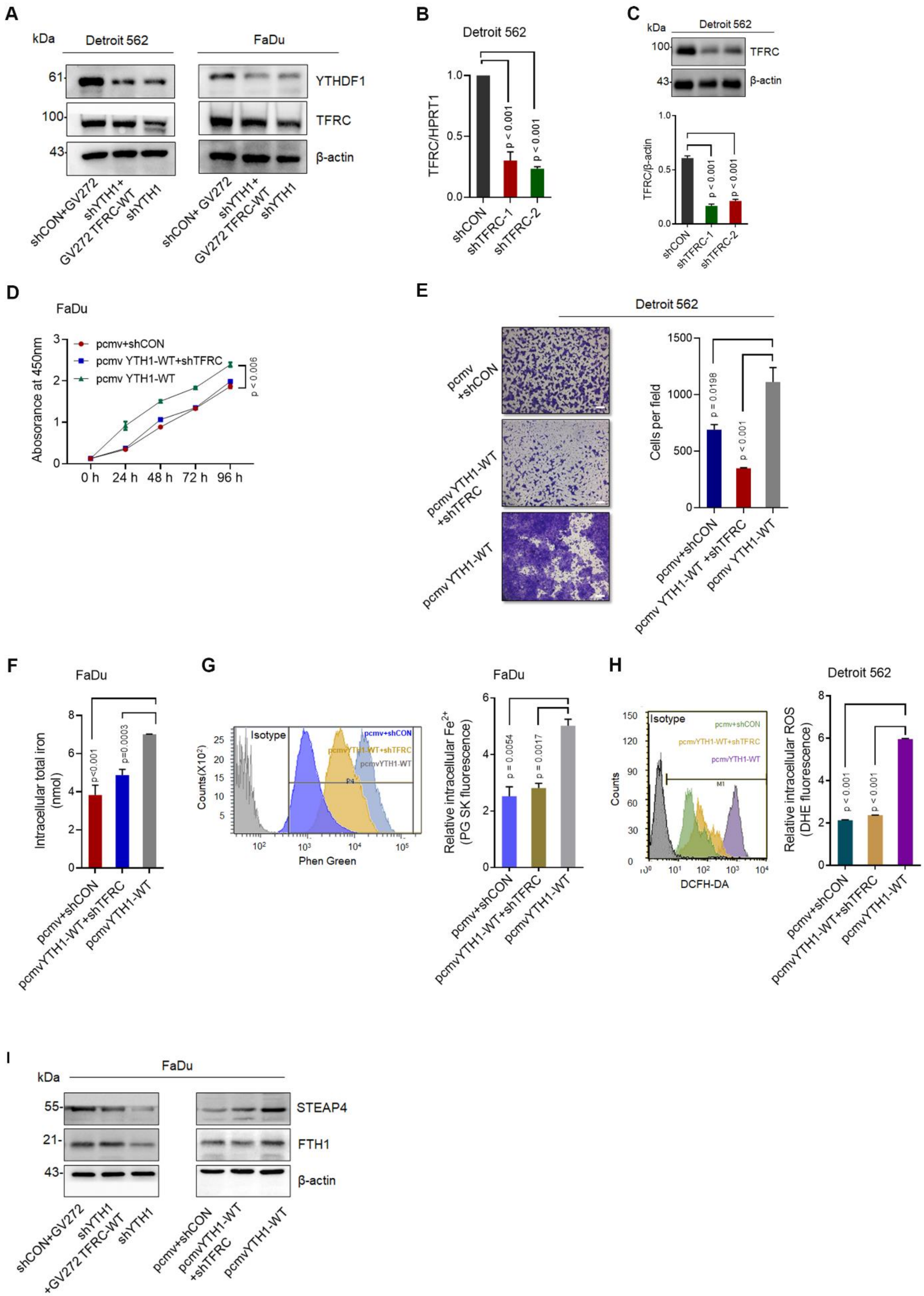

A
kDa
Detroit 562
FaDu
61
100
43
YTHDF1
TFRC
β-actin
shCON+GV272
shYTH1+ GV272 TFRC-WT
shYTH1
shCON+ GV272
shYTH1+ GV272 TFRC-WT
shYTH1
B
Detroit 562
TFRC/HPRT1
p < 0.001
p < 0.001
shCON
shTFRC-1
shTFRC-2
C
kDa
Detroit 562
100
43
TFRC
β-actin
TFRC/β-actin
p < 0.001
p < 0.001
shCON
shTFRC-1
shTFRC-2
D
FaDu
Abosorance at 450nm
pcmv+shCON
pcmv YTH1-WT+shTFRC
pcmv YTH1-WT
p < 0.006
0 h
24 h
48 h
72 h
96 h
E
Detroit 562
pcmv +shCON
pcmvYTH1-WT +shTFRC
pcmvYTH1-WT
Cells per field
p = 0.0198
p < 0.001
pcmv+shCON
pcmv YTH1-WT +shTFRC
pcmv YTH1-WT
F
FaDu
Intracellular total iron (nmol)
p<0.001
p=0.0003
pcmv+shCON
pcmvYTH1-WT+shTFRC
pcmvYTH1-WT
G
Isotype
pcmv+shCON
pcmvYTH1-WT+shTFRC
pcmvYTH1-WT
Counts(X10²)
Phen Green
FaDu
Relative intracellular Fe²⁺ (PG SK fluorescence)
p = 0.0054
p = 0.0017
pcmv+shCON
pcmvYTH1-WT+shTFRC
pcmvYTH1-WT
H
Isotype
pcmv+shCON
pcmvYTH1-WT+shTFRC
pcmvYTH1-WT
M1
Counts
DCFH-DA
Detroit 562
Relative intracellular ROS (DHE fluorescence)
p < 0.001
p < 0.001
pcmv+shCON
pcmvYTH1-WT+shTFRC
pcmvYTH1-WT
I
kDa
FaDu
55
21
43
STEAP4
FTH1
β-actin
shCON+GV272
shYTH1 +GV272 TFRC-WT
shYTH1
pcmv+shCON
pcmvYTH1-WT +shTFRC
pcmvYTH1-WT

Figure S5

A. Validation of TFRC overexpression efficiency in Detroit 562 and FaDu HPSCC cells as assessed by Western blot.

B, C. Validation of TFRC shRNA efficiency as assed by qPCR (B) and Western blot analysis (C).

D, E. CCK-8 (D) and Transwell assays (E) of different groups. Magnification: E, 5 ×, scale bar = 100 μm.

F. Intracellular iron levels (nmol) were measured in FaDu cells after different treatments. G, H. Flow cytometry and quantification results based on analysis of the Phen Green- and LDH-positive cells indicating intracellular Fe^{2+} levels (G) and ROS levels (H) in FaDu cells after different treatments.

I. Western blot analysis of the indicated proteins in FaDu cells after different treatments.

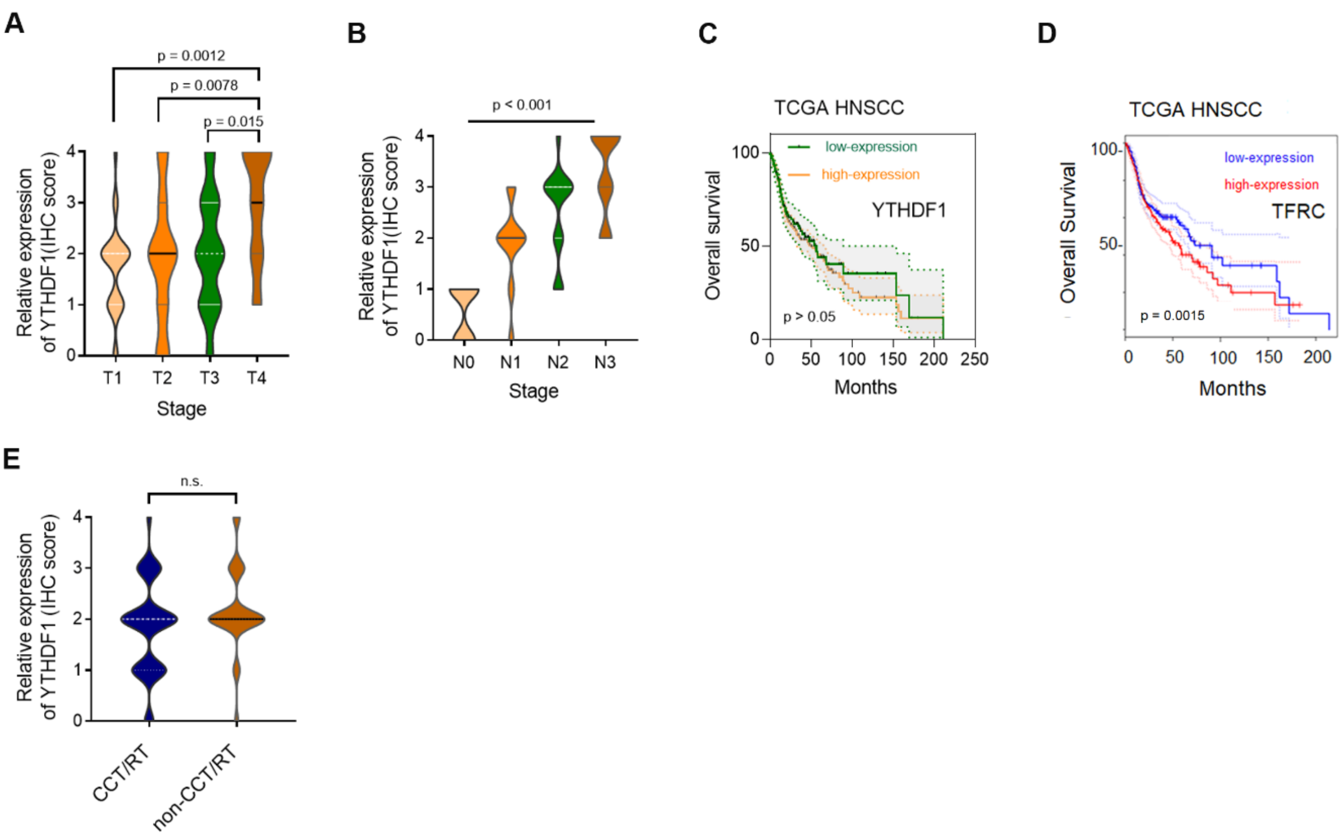

Figure S6

A, B. Relative expression of YTHDF1 in HPSCC at different T stages (A) and N stages (B).

C, D. Kaplan–Meier analysis of HNSCC patients based on the correlations between YTHDF1 and TFRC expression and recurrence-free survival; data were generated with the TCGA head and neck dataset.

E. Statistical analysis of the relative expression of YTHDF1 (IHC score) in HPSCC patients who received or did not receive CCT/RT, as ascertained by the Mann-Whitney U test

Supplementary data 1 :HPSCC patients in cohort 1 and cohort 2

Supplementary data 2 : Original M^6A peak, RIP data, overlapping M^6A peaks and RIP data. xlsx

Supplementary data 3: Primer list and mutation sequences. Xlsx

Supplementary data 4: Supplementary Materials and Methods

Patient specimens

Two Cohorts of HPSCC patients from individual medical centers who underwent surgery and regular medical surveillance between 2012 and 2020 were used in this study.
Cohort1 (Blood, fresh cancerous and normal tissues, and paraffin-embedded surgical specimen) were from Sir Run Run Shaw Hospital, College of Medicine, Zhejiang University. Blood, fresh cancerous and normal tissues were stored and numbered in Sir Run Run Shaw hospital biological specimen Bank. Paraffin-embedded surgical specimen was stored permanently in Sir Run Run Shaw hospital pathology department.
T2- or T2*-weighted images of Magnetic Resonance Imaging(MRI) enable qualitative assessment of lesion iron. Images show representative T2 weighted images in patients with different levels of iron overload. Several quantitative MR-based strategies have been employed to measure LIC: signal intensity ratio (SIR) techniques based on T2-weighted or T2*-weighted imaging, quantitative relaxometry (largely R2- and R2*-based), and MR susceptometry. R2 and R2* values generated comparable noninvasive estimates of intratumor iron concentration(IC) in a study by Wood et al[1].
Cohort 2 (paraffin-embedded surgical specimen) were from The Second Affiliated Hospital of Nanchang University Medical College. None of these patients had received radiotherapy or chemotherapy prior to surgery. None of them had hepatic disease.

Global m^{6}A measurement

The global m^{6}A levels in mRNA were measured with EpiQuik m^{6}A RNA Methylation Quantification Kit (Colorimetric) (Epigentek, Farmingdale, NY) following the manufacturer's protocol.

Transcriptome-Seq and M^{6}A-seq

Total RNA was extracted using Trizol reagent (Invitrogen, CA, USA) following the manufacturer's procedure. The total RNA quality and quantity were analysis of Bioanalyzer 2100 and RNA 6000 Nano LabChip Kit (Agilent, CA, USA) with RIN number >7.0. Approximately more than 200 ug of total RNA was subjected to isolate Poly (A) mRNA with poly-T oligo attached magnetic beads (Invitrogen). Following purification, the poly(A) mRNA fractions is fragmented into ~100-nt-long oligonucleotides using divalent cations under elevated temperature. Then the cleaved RNA fragments were subjected to incubated for 2h at 4℃ with m^{6}A-specific antibody (No. 202003, Synaptic Systems, Germany) in IP buffer (50 mM Tris-HCl, 750 mM NaCl and 0.5% Igepal CA-630) supplemented with BSA (0.5 μg μl−1). The mixture was then incubated with protein-A beads and eluted with elution buffer (1 × IP buffer and 6.7mM m^{6}A). Eluted RNA was precipitated by 75% ethanol. Eluted m^{6}A-containing fragments (IP) and untreated input control fragments are converted to final cDNA library in accordance with a strand-specific library preparation by dUTP method. The average insert size for the paired-end libraries was ~100±50 bp. And then we performed the paired-end 2×150bp sequencing on an Illumina Novaseq™ 6000 platform at the LC-BIO Bio-tech ltd (Hangzhou, China) following the vendor's recommended protocol.
For m^{6}A sites prediction: By applying the HOMER motif discovery tool, we found that the "GGAC" consensus sequence was the common m^{6}A motif enriched in the m^{6}A peaks. Furthermore, most of the YTHDF1-binding sites were highly enriched in 5'UTR and 3'UTR, especially enriched in the vicinity of the stop codon, which was coincidence with the m^{6}A distribution. We then download the DNA sequences on Ensembl (www.ensembl.org).GGACU, GGACA, GGACC, AGACU were input to search for sequences consistent with RRACH motif rules. Then A was replaced with T. The detailed mutation sequences are in Supplementary data

3.

Anti-m6A immunoprecipitation and RNA immunoprecipitation(RIP)

Cells were washed twice with PBS, collected and then the pellet was resuspended in IP lysis buffer (150mM KCl, 25mM Tris (pH 7.4), 5mM EDTA, 0.5mM DTT, 0.5% NP40, 1× protease inhibitor, 1 U/μl RNase inhibitor).The lysate was harvested by centrifugation at 12 000 g for 10 min after incubation for 30 min. Antibodies and 40μl of protein G beads (Invitrogen, USA) were added into the lysate followed by incubation overnight at 4℃. After washed three times with wash buffer (150mM KCl, 25mM Tris (pH 7.4), 5mM EDTA, 0.5mM DTT, 0.5% NP40), co-precipitated RNAs were extracted by Trizol reagent, ethanol-precipitated with glycogen (Invitrogen, USA). The enrichment of RNAs was normalized to IgG. For sequencing, rRNAs was depleted by using the NEBNext rRNA depletion kit (New England BioLabs). cDNA libraries were produced by employing NEBNext UltraRNALibrary Prep Kit for Illumina (New England BioLabs) and sequenced on Illumina Novaseq™ 6000 platform at the LC-BIO Bio-tech ltd (Hangzhou, China) following the vendor's recommended protocol. TFRC mRNA levels in the elutes were measured by RT-PCR with primers.

Constructs and cell culture

HPSCC Detroit562 (ATCC@ CCL138™) and FaDu (ATCC@ HTB-43™) cells were purchased from American type culture collection (Manassas, VA) in 2017. Detroit 562 is a metastatic pharyngeal SCC cell line which was obtained from the hydrothorax. FaDu is a primary hypopharyngeal SCC cell line. They both exhibit highly invasive behavior *in vivo*. The cell line was maintained in complete media of dulbecco's modified eagle medium (DMEM) (Solarbio, China) added 10 % bovine serum albumin, 1 % penicillin-streptomycin (Solarbio, China) in an incubator with 5 % CO2, 37℃.

Lentivirus and Plasmid construction

shRNA against YTHDF1 and two independent shRNA against TFRC were constructed using GV248 vector. YTHDF1-WT and YTHDF1-Mut (NM_017798 K395A, Y397A) was amplified by PCR and cloned into CMV6-MCS-3flag-SV40-Neomycin vector. For xenograft model YTHDF1-WT and YTHDF1-Mut (NM_017798 K395A, Y397A) were constructed using GV115 vector. TFRC-WT and TFRC-Mut was amplified by PCR and cloned into GV272 vector. Those recombinant plasmids were constructed by Gene Chemistry(Shanghai, China). All the constructs were verified by sequencing, detailed cloning information can be provided upon request.

Lentivirus packaging and generation of stable cell lines

The lenti-viruses were generated according to the manufacturers protocol. Lipofectamine 3000 reagent (Invitrogen) was incubated with Opti-MEM I Reduced Serum Medium (GIBCO), and HEK239T cells were transfected with 7.5ug of plasmid. Eight hours after transfection, the medium was replaced with 10 mL of fresh medium. The supernatant containing the lentivirus particles were collected 48h post-transfection, filtered through 0.45μm PVDF filter and transfected into cells immediately. Viruses carrying a given plasmid were premixed 1:1, and 50 μl of virus was added to 1 mL of serum. 24 hours prior to transfection, tumor cells were seeded at 1.0 $\times 10^5$ cells per well in 6 cm dishes, and the medium was replaced with virus-containing supernatant. After 48 h, the medium was replaced with fresh medium. Cells were selected by incubation with 4 μg/ml puromycin for 2 weeks and maintained in 1 μg/ml puromycin.

Cell growth, cell proliferation, migration

Cell viability was detected by adding 10% CCK8 (DOJINDO, Japan) into the infected cells

plated in 96-well plates and incubation at 37℃ for 30min-45min at 0, 24, 48, 72 and 96 h. The absorbance of each well was measured by a microplate reader set at 450 nm. For colony formity assays, tested cell lines were plated into 12-well plates and the cell numbers were subsequently counted each day.

For cell migration assays, cell lines were plated into a 24-well Transwell chamber system (Corning, USA). 15 × 10^4 cells were seeded in the upper chamber of an insert with 0.4 ml serum-free culture media in 24-well plates. 0.6 ml culture media with 20% FBS were added to the lower chamber. After incubation for 24 h, cells were fixed in 4% paraformaldehyde for 20 min and then stained with 0.1% crystal violet (Sigma-Aldrich, USA) for 30 min. Random fields were photographed and the number of migrated cells was determined.

Iron assay, Intracellular chelatable iron (Fe^{2+}) and ROS measurement

Intracellular iron (ferrous iron and ferric iron) level was determined using the iron assay kit from Sigma-Aldrich (MAK025). Intracellular chelatable iron (Fe^{2+}) was tested by the fluorescent indicator Phen Green SK fluorescent probe (#P-14313, Life Technologies, Grand Island, NY, USA). Reactive oxygen species (ROS) was determined using DCFH-DA (Solarbio, D6470) according to the manufacture introduction. According to the manufacture introduction, HPSCC cells were incubated with Phen Green SK or Dihydroethidium(DHE) for 20min under 37℃. Excess dye was removed by washing twice with PBS. Labeled cells were then trypsinized and resuspended in PBS and transferred to a 5 ml FACS tube. Analysis within 10 min on flow cytometer (BD FACS Calibur). For PG SK fluorescence measurements (excitation maximum,507 nm; emission maximum, 532 nm). For DHE(excitation maximum 530 nm; emission maximum 610 nm). The ratio of Mean fluorescent Intensity (MFI) was calculated for each sample. The data were normalized to control samples as shown by the relative Fe^{2+} or ROS.

Histology, immunohistochemical staining and immunofluorescence staining

Paraffin-embedded tissue sections (5 μm) were deparaffinized in xylene and rehydrated in ethanol gradient. Sections were also stained with hematoxylin and eosin (HE). For immunohistochemical (IHC) staining, the slides were incubated with 10% preimmune serum for 60min at room temperature. After incubation with a 1:100 dilution of primary antibody to YTHDF1(Abcam: ab220162), to TFRC(Abcam: ab214039) at 4 ℃ overnight, slides were rinsed with phosphate-buffered saline (PBS) and incubated with a labeled polymer-HRP (GTVison TMIII, GK500710) incubated for 60 min. Color reaction was developed by using 3, 3'-diaminobenzidine tetrachloride (DAB) chromogen solution. All slides were counterstained with hematoxylin. The matched IgG isotype antibody was used as a negative control. Staining intensity for YTHDF1 and TFRC were scored as 0 (negative), 1 (weak), 2 (moderate), and 3 (strong). Staining extent was scored as 0 (0%), 1 (1-25%), 2 (26-50%), 3 (51-75%), and 4 (76-100%). The scores for IHC staining of tissue microarrays sections were characterized independently by two pathologists, at 5X and 40 X magnification based on the staining intensity and extent of staining. All the p values were based on the two-sided statistical analysis and P-value less than 0.05 was statistically significant.

Total RNA extraction and quantitative real-time PCR(qPCR)

Total RNA was extracted from HPSCC tissues and cells using Trizol (Invitrogen, USA) according to the manufacturer's instruction and quantified by Nanodrop. A total of 500 ng RNA was reverse transcribed using Reverse Transcription Kit (Takara, Dalian, China) and was then subjected to qPCR with SYBR green master mix (Invitrogen) using recommended conditions in the LC480 (ROCHE). The $2^{-\Delta\Delta Ct}$ method was used to quantify the relative expression levels. HPRT1 was used as an internal control. Primers are listed in **Supplementary data 3.**

Western Blot Analysis

Western blot analysis was performed as previously described[2]. The antibodies against

YTHDF1(Abcam: ab220162), to Ferritin(Abcam: ab199256) , to TFRC(Abcam: ab214039), to FTH1 (Abcam: 81444), to STEAP4 (11944-1-AP; Proteintech).

Mouse treatment and tumor biology studies

HPSCC FaDu or Detroit 562 cell lines (1×10^6 cells) stably expressing vector control and construct lentivirus were injected subcutaneously into the right flank of nude mice. Tumor sizes and mice weight in all groups were measured every 3 days for 1-6 weeks. For DFP treatment, male nude mice at 4 weeks of age were injected subcutaneously into the right flank with Detroit 562 and FaDu cell lines (1×10^6 cells). After 12 days, mice were divided into control (H_2O) and DFP groups (n=5 per group). DFP concentration: 1 mg/mL in the drinking water in for 12 days.

All mice were maintained in standard cages in a light and temperature-controlled room and were given standard chow and water. All animal studies were carried out in accordance with Institute of Laboratory Animal Resources guidelines and approved by the University Committee on the Use and Care of Animals at the Zhejiang University.

Polysome profiling.

Based on the documented procedure, we started with nine 15-cm dish of indicated cells. Before collection, cycloheximide (100 μg/ml) was added into cell culture media for 5 min. Cells were collected, washed and lysated with lysis buffer: 5 mM Tris-HCl (pH 7.5), 2.5mM MgCl2, 1.5mM KCl, 100 μg/ml cycloheximide, 2mM DTT, 0.5% Triton X-100, 0.5% Sodium Deoxycholate, 200U/ml RNase inhibitor, 1x protease inhibitor cocktail (EDTA-free). The cell supernatants were fractioned (total 72 fractions, 0.45 ml per fraction), and then analyzed by NanoDrop (Thermo Fisher Scientific) for OD260. Sample from each fraction was subjected to Real-time RT-PCR analysis of relative mRNA expressions of genes.

Luciferase assay

The fragments of TFRC-5'UTR, and TFRC -3'UTR containing the wild-type m6A motifs as well as mutant m6A motifs (m6A was replaced by T) were synthesized at Gene Chemistry(Shanghai, China). The firefly luciferase and Renilla luciferase activities in each well were calculated by a dual-luciferase reporter assay system (GV272). The ratios between the 5'UTR and 3′UTR reporter of TFRC and Renilla control were determined 48 h after siRNA treatment. The relative luciferase activity was further normalized to that in cells transfected with the firefly luciferase vector control under the same treating conditions. Firefly luciferase activity and Renilla luciferase activity were measured using FLUOstar Omega (BMG LABTECH, Offenburg, Germany). The results were shown in the form of relative firefly luciferase activity normalized to Renilla luciferase activity. All the experiments were repeated for three times, and three replicates were conducted for each group.

Cellular Labile Iron Pool Quantification

The labile iron pool (LIP) was visualized using the fluorescent dye Calcein-AM as previously described with modifications (Epsztejn et al., 1997). Briefly, after sample treatment, cells were trypsinized, spun at 1,200 rpm for 5 min, and resuspended at approximately 1 × 106 cells mL−1 in 500 nM Calcein-AM in PBS. Samples were incubated for 15 min at 4% O2 in a humidity controlled environment (37 °C, 5% CO 2). Subsequently, samples were pelleted, washed in PBS, and resuspended in 1 mL PBS before dividing each sample into two flow cytometry sample tubes, to which 100 μM 2′,2′-bipyridyl (BIP) was added to one tube. Samples were kept at room temperature and 10,000 cells were analyzed on a LSR II Flow Cytometer (BD Biosciences; λex = 488 nm, λem = 515/20 nm). BIP tubes were incubated for at least 15

minutes before analysis to allow for full chelation of intracellular labile iron. The LIP (A.U.) = MFIBIP – MFINoBIP was normalized against the control samples to calculate the relative labile iron pool.

Protein stability

To evaluate protein stability, FaDu cells were treated with 100 μg/ml protein translation inhibitor cycloheximide (CHX) during indicated times and harvested. Then protein expression of TFRC was determined by Western Blot analysis.

Statistics methods

Bioinformatic analysis including GO, KEGG, GSEA analyses were performed using the OmicStudio tools at https://www.omicstudio.cn/tool. All statistical analysis was carried out using GraphPad Prism version 7 (GraphPad Software, CA) for Windows or the program R (www.r-project.org). R code are listed in **Supplementary data 5.** Statistical significance was assessed by unpaired two tailed Student's-tests, analysis of variance tests (ANOVA) or Spearman rank correlation. Recurrence-free survival was evaluated by Kaplan-Meier survival curve and Log-rank tests. The data are expressed as mean ±SD. *P < 0.05; **P < 0.01; ***P < 0.001. Each experiment was repeated independently at least three times.

Reference:

1. Wood JC, Enriquez C, Ghugre N, Tyzka JM, Carson S, Nelson MD, Coates TD: **MRI R2 and R2* mapping accurately estimates hepatic iron concentration in transfusion-dependent thalassemia and sickle cell disease patients.** *Blood* 2005, **106:**1460-1465.
2. Ye J, Jiang X, Dong Z, Hu S, Xiao M: **Low-Concentration PTX And RSL3 Inhibits Tumor Cell Growth Synergistically By Inducing Ferroptosis In Mutant p53 Hypopharyngeal Squamous Carcinoma.** *Cancer Manag Res* 2019, **11:**9783-9792.

Supplementary data 5: R code used for data analysis
